# Prevalent pH Controls the Capacity of *Galdieria maxima* to Use Ammonia and Nitrate as a Nitrogen Source

**DOI:** 10.3390/plants9020232

**Published:** 2020-02-11

**Authors:** Manuela Iovinella, Dora Allegra Carbone, Diana Cioppa, Seth J. Davis, Michele Innangi, Sabrina Esposito, Claudia Ciniglia

**Affiliations:** 1 Department of Biology, University of York, York YO105DD, UK; mi676@york.ac.uk (M.I.); seth.davis@york.ac.uk (S.J.D.); 2 Department of Biology, University of Naples Federico II, 80126 Naples, Italy; doraallegracarbone@gmail.com (D.A.C.); dianacioppa@gmail.com (D.C.); 3 Key Laboratory of Plant Stress Biology, School of Life Sciences, Henan University, Kaifeng 475004, China; 4 Department of Environmental, Biological and Pharmaceutical Science and Technology, University of Campania “L. Vanvitelli”, 81100 Caserta, Italy; michele.innangi@unicampania.it (M.I.); sabrina.esposito@unicampania.it (S.E.)

**Keywords:** *Galdieria*, pH, ammonium, nitrate

## Abstract

*Galdieria maxima* is a polyextremophilic alga capable of diverse metabolic processes. Ammonia is widely used in culture media typical of laboratory growth. Recent reports that this species can grow on wastes promote the concept that *G. maxima* might have biotechnological utility. Accordingly, there is a need to know the range of pH levels that can support *G. maxima* growth in a given nitrogen source. Here, we examined the combined effect of pH and nitrate/ammonium source on the growth and long-term response of the photochemical process to a pH gradient in different *G. maxima* strains. All were able to use differing nitrogen sources, despite both the growth rate and photochemical activity were significantly affected by the combination with the pH. All strains acidified the NH_4_^+^-medium (pH < 3) except *G. maxima* IPPAS P507. Under nitrate at pH ≥ 6.5, no strain was able to acidify the medium; noteworthy, *G. maxima* ACUF551 showed a good growth performance under nitrate at pH 5, despite the alkalization of the medium.

## 1. Introduction

Polyextremophiles are organisms able to survive and grow under more than one harsh environmental conditions, such as extremes of temperature (thermophily), pH (acidophily, alkaliphyles), salts (halophily), and pressure (barophily) [1,2]. The ability to counteract environmental stressors is related to the development of specific mechanisms concerning structural and biochemical adaptation, gene expression, and regulation. These make such organisms essential sources for biotechnological applications [1].

Geothermal environments, characterized by high temperatures (above 50 °C), low pH values (<1), and high amounts of heavy metals, are common areas where polyextremophiles are undisputed rulers: Bacteria, fungi, and some algae are the only surviving microorganisms [3,4]. Among the latter, Cyanidiophyceae (Rhodophyta) represents almost all the thermoacidophilic eukaryotic biomass [5]. Cyanidiophyceae (*Galdieria sulphuraria*, *G. maxima*, *G. phlegrea, Cyanidium caldarium*, and *Cyanidioschyzon merolae*) are the most abundant photosynthetic protists found in extremely acidic, sulfur-rich environments generally bordering active volcanoes [6,7,8,9,10,11,12,13,14]. Condensation of sulfur dioxide and hydrogen sulfide produces sulfur crystals, which are subsequently oxidized to sulfuric acid resulting in acidification (pH 0.5–3). Sulfur allows cells to produce several structural and functional compounds such as amino acids cysteine and methionine, glutathione, sulpholipids, and cell wall constituents [3,15,16].

Recent genome sequencing demonstrated that horizontal gene transfer (HGT) from prokaryotic sources provided *G. sulphuraria* extraordinary metabolic flexibility. It is able to use more than 50 different carbon sources [17,18,19,20,21] and to survive in hostile environments rich in heavy and rare metals (e.g., genes to detoxify mercury and arsenic). The extent of metabolic flexibility for other elements, such as prevailing N is less explored, but it is known that amino acids can be used as an N-growth component.

Thus far, Cyanidia was isolated from several solfataras worldwide (Italy, Iceland, Japan, New Zealand, Yellowstone National Park, and Taiwan). The acidophily was considered as one of the main factors limiting the dispersal and survival of Cyanidiophyceae; nevertheless, recent explorations in Turkey provided the ubiquity of Cyanidia in several Turkish thermal baths, mostly on neutral and sub neutral soils (pH 5.8–7), growing as thin biofilms hypolithically and endolithically [13]. The ability to lower the external pH from 6 to more acidic values was tested by Lowell and Castenholtz (2013) on several Cyanidiophiceae strains collected from Yellowstone, Japan, Philippines, and New Zealand hot springs, suggesting the importance of this process as a survival strategy in nonacidic environments during long-distance migration [3,20,22]. Growth in the sub-neutral medium was also evaluated by Henkanatte-Gedera et al. (2017), both at the laboratory and field scale. These data confirmed the capacity of *G. sulphuraria* to grow in a broader range of initial environmental pHs, suggesting the potential savings in chemical costs associated with pH adjustment of real waste streams [23].

The employment of *Galdieria* species in applied sciences prompted us to assess the effect of initial pH on the growth of eight *G. maxima* strains collected from acidic, neutral, and sub-neutral thermal areas. Likewise, since proton exchange can be dependent on the uptake and metabolism of nitrogen compounds [24], we investigated the combined effect of pH and nitrate/ammonium source on their growth. We also evaluated the long-term response of the photochemical process to a pH gradient, considering the maximum efficiency of photosystem II (Fv/Fm) and the non-photochemical quenching (NPQ) as indicators of the physiological state of the cultures as a response to pH and nitrogen source. 

## 2. Material and Methods

### 2.1. Strains Cultivation

Eight *Galdieria maxima* strains, supplied from the Algal Collection of University Federico II of Naples (www.acuf.net) and formerly collected from Turkey [13], Iceland [9,12], and Russia [16], were selected based on the pH of the original collection site (Table 1). All strains were isolated by streaking across the agar plates, and colonies were inoculated in an Allen medium containing (NH_4_)_2_SO_4_ as the nitrogen source, at pH 1.5 [25] and cultivated at 42 °C under continuous fluorescent illumination of 50 µmol photons·m^−2^·s^−1^ until they reached the exponential phase. 

### 2.2. Phylogenetic Analysis

We retrieved from GenBank 197 *rbcL* nucleotide sequences of RuBisCO large subunit gene (*rbcL*) from different Cyanidiophyceae species and strains [6,7,8,9,12,13]; *G. maxima* ACUF551 was lacking in the database. Thus DNA extraction was performed using the protocol described in Ciniglia et al., 2015 [26]. Four degenerate primers were used to amplify the *rbcL* gene from the sample [6]. The resultant product was purified with the QIAquick PCR purification kit (Qiagen) and used for direct sequencing using the BigDyeTM Terminator Cycle Sequencing Kit 3.1 (PE- Applied Biosystems, Norwalk, CT, USA) and an ABI-3500 XL at the Microgem Laboratory (Naples, Italy). Forward and reverse electropherograms were assembled and edited using the program Chromas Lite v.2.1 (http://www.technelsium.com.au/chromas.html). All sequences were aligned with the MUSCLE command in UGENE v 1.16.0 [27,28]; 4 species belonging to the subphylum Rhodellophytina (*Rhodella violacea*, *Dixoniella grisea*, *Porphyridium aerugineum*, *Bangiopsis subsimplex*) Rhodophyta, were chosen as the outgroup (Table S1). No gaps or indels were incorporated in the alignment, and the best likelihood tree was estimated under the general time-reversible (GTR) substitution with gamma-distributed rate heterogeneity (G) and proportion of invariable sites estimation (I) as a result of model-testing in jModeltest v.2.1.10 [29]. The maximum likelihood (ML) analysis was performed with RAxML v.8.2.8 using the rapid bootstrap option (1000 replicates) to evaluate the branches’ statistical support [30].

### 2.3. Experimental Procedure

Microalgal cultures (OD~0.35; 1.5-2.0•10^7^ cells) were inoculated in fresh Allen medium under different pH levels (pH 5, 6, 6.5, and 7) to investigate the influence of pH on growth; control tests were performed at pH 1.5, that is considered as optimal for Cyanidiophyceae. These experiments were carried out in 50ml Erlenmeyer flasks containing Allen medium with (NH_4_)_2_SO_4_ as the nitrogen source (10 mM) at 42 °C under continuous cool fluorescent light (50 µmol photons·m^−2^·s^−1^) and kept mixed on an orbital shaker illuminated beneath. All analyses were performed in triplicate.

The pH of the medium was measured weekly for 6 weeks (ph meter Mettler, Toledo), and the growth rates were monitored by spectrophotometric measurements of the optical density OD_550_ (Secomam spectrophotometer Prim light). The effect of the pH on the growth of the algal strains was assessed by calculating the maximum growth rate using the equation:

$${\mathrm{RGR}}_{\left(1/d\right)}=\frac{\mathrm{Ln}\left(\mathrm{Nt}\right)-\mathrm{Ln}\left(N_{0}\right)}{\left(t-t_{0}\right)}$$

where: Nt is the optical density at the final time 

N_0_ is the optical density at the initial time 

T is the final time (days)

T_0_ is the initial time (days)

The effect of different nitrogen source was next evaluated on 3 strains (IPPAS P507, ACUF722, and ACUF551), selected based on the different pH responses; the algal strains were grown in Allen medium where the ammonium was replaced by nitrate (NaNO_3_) in an equimolar quantity, at pH 7, 6.5, 5, and 1.5, using the same experimental design of previous experiments. In these assays, tests were also performed in Allen medium containing ammonium at the same pHs. Chlorophyll a content (AquaFluorTM; Handheld Fluorometer/Turbidimeter; Turner Designs) and pH variations were recorded at 4-day intervals during the experiments for 12 days. All measurements were taken in triplicates.

The photochemical activity of IPPAS P507, ACUF722, and ACUF551 was determined with PAM Fluorometry [31] at 42 °C in a thermostatic chamber (Hansatech) by pulse amplitude modulated fluorometry (FMS-2 pulse-modulated chlorophyll fluorometer, Hansatech Instruments Ltd., UK), following the Hanlet protocol for red algae [32]. After a dark period (30 min), 2 mL of each sample were placed in a quartz cuvette near the optic fiber of the fluorimeter, under continuous stirring. During the analyses, microalgae were exposed at four different light intensity (31 μmol photons m^−2^ s^−1^, 65 μmol photons m^−2^ s^−1^, 116 μmol photons m^−2^ s^−1^, and 281 μmol photons m^−2^ s^−1^), alternating three minutes of light and three minutes of dark [33,34]. Three variables were considered, the maximum efficiency of photosystem II, non-photochemical quenching, and quantum yield of photosystem II. The maximum efficiency of photosystem II (Fv/Fm), index of the relatively good physiological state of the cultures, was evaluated in the dark with a saturating light pulse of 2900 μmol photons m^−2^ s^−1^ at 0.6s. Non-photochemical quenching (NPQ) was determined by the formula (Fm–F0)/F0 and used as an indication of algal stress conditions [35]. The quantum yield of photosystem II (ΦPSII) is an indicator of PSII photochemical efficiency in the presence of light. All measurements were done in triplicates.

### 2.4. Statistical Analysis

All experiments were carried out in triplicate, and results were reported as mean ± standard deviation. Variables were inspected for normality and homogeneity of variance. Variations of the relative growth rate, culture medium pH, chlorophyll content, and photochemical activity were tested by means of a one-way analysis of variance (one-way ANOVA). As a multiple comparison post-hoc test to evaluate the significance of differences among treatments, a Tukey HSD was used (α = 0.05). All statistical analyses were performed with R studio software [36].

## 3. Results

### 3.1. Phylogenetic Analysis

**Figure 1:**
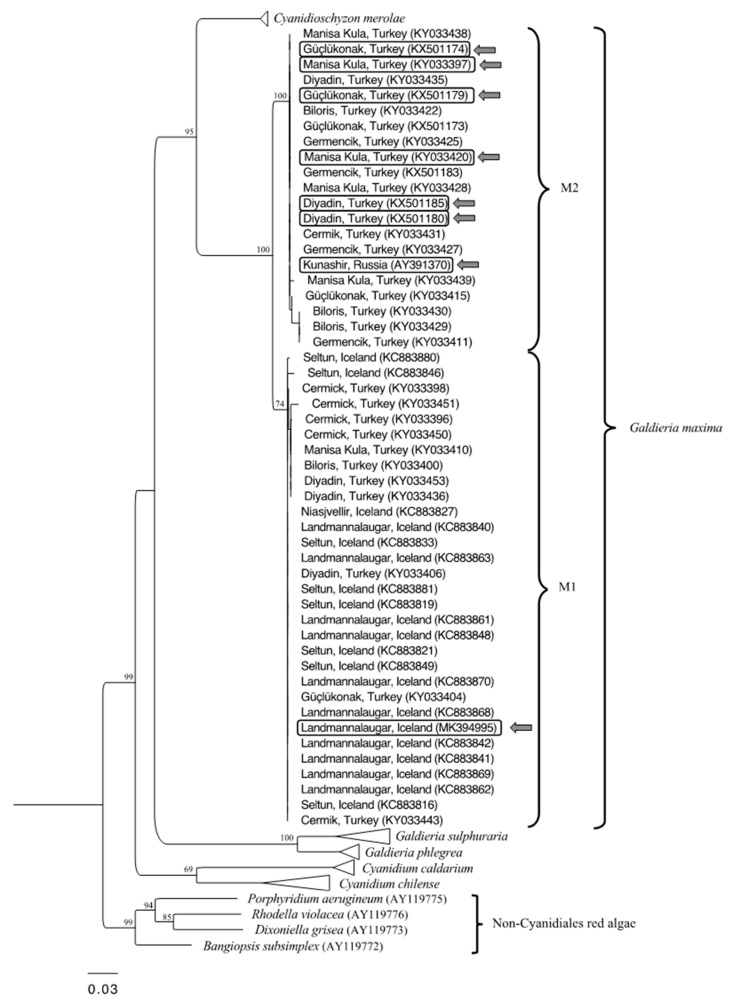
Maximum likelihood tree of Cyanidiophyceae analysis based on rbcL sequences. Only bootstrap values > 60% are shown near the nodes. The arrows indicate the G. maxima strains selected for the experiments. See Table S1 for details.

Molecular characterization, based on the partial (842 bp) *rbcL* phylogeny, classified all the isolates chosen for these experiments as *G. maxima* (Tested strains were circled and indicated from the arrows; Figure 1). The analysis identified a divergence of *G. maxima* in two subclades (100% MLB), and the grouping of most of Turkish *G. maxima* isolates in the sub-population GM2 (100% MLB). All these isolates shared 100% of identical sites with the Russian strain IPPAS P507 from the Kamchatka peninsula. The other subclade (GM1) comprised other Turkish strains and the ones collected from the Icelandic areas (74% MLB). The *rbcL* sequence of the Icelandic isolate ACUF551, belonging to this group, slightly diverged from the reference sequence (IPPAS P507), sharing with it 95.6% of identical sites.

### 3.2. Relationship between pH and Growth under Ammonium Source

**Figure 2:**
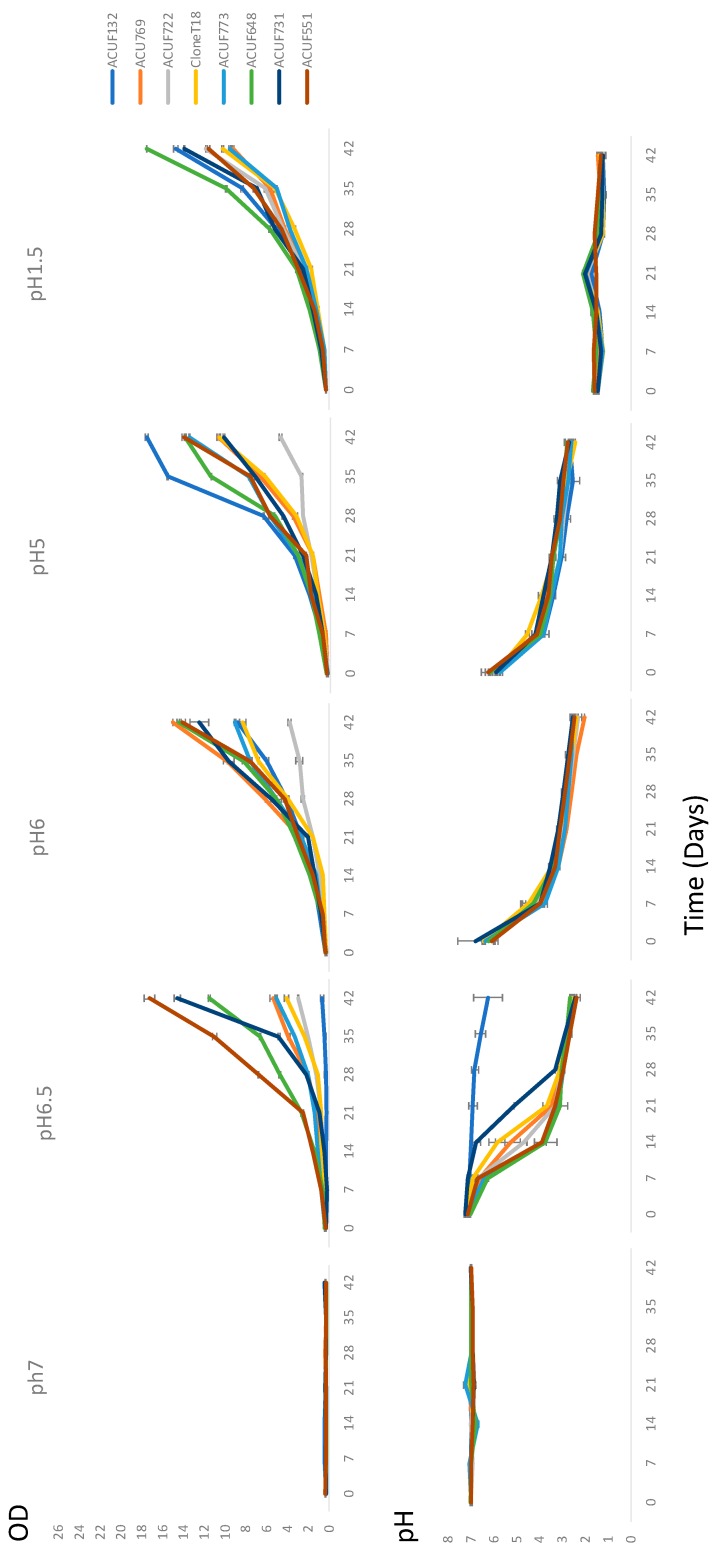
The trend of the pH and growth as a function of time for the experiments starting from pH 7, 6.5, 6, 5, and 1.5. The analysis of variance was carried out by a one-way Anova (*p* < 0.05). Differences between treated and control samples were evaluated by Dunnet’s test; optical densities (OD) were significantly different from the control level mean for treatments at initial pH > 6.5; pHs were significantly different from the control level mean for treatments at initial pH > 5.

The growth of 8 *G. maxima* strains was followed and related to the lowering of pH for 6 weeks, starting from different pHs and providing NH_4_^+^ as the nitrogen source. At an initial pH 1.5, the external pH remained stable, with low and non-significant variations among all strains and throughout the experiments. In contrast, when considering a starting pH 7, none of the strains displayed detectable growth after the 6 weeks, and small variations of pH were recorded in all cultures (Figure 2, Table S2). 

**Figure 3:**
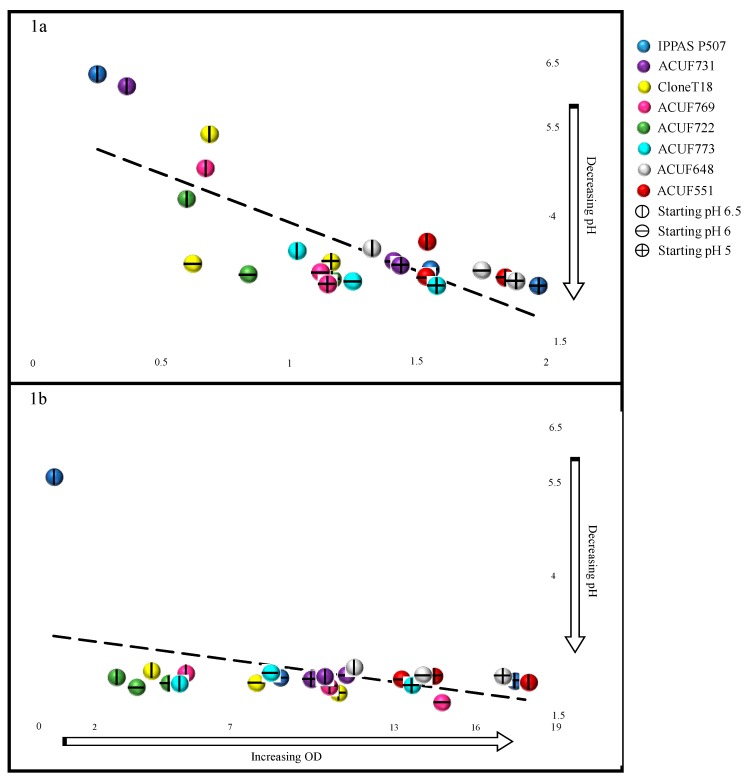
Relationship between pH (y-axis) and growth rate (x-axis) for all the strains after 2 weeks (**1a**) and 6 weeks (**1b**). correlation = −0.75 (2 weeks), −0.43 (6 weeks).

Plotting the concurrent lowering of external pH with the increase of biomass yield during the experiments started from pH 5, 6, and 6.5, a correlation existed between the final pH and the biomass yield after 2 (Figure 3a) and 6 weeks (Figure 3b). The graphs were divided into quadrants based on the growth and pH. 

A significant correlation was recorded between the pH and growth rate after 2 weeks (corr = −0.75; *P* < 0.05). Neither IPPAS P507 nor ACUF731 grew (OD < 0.5) or a lowered pH of the medium at a starting pH 6.5. Strains ACUF722, ACUF769, and CloneT18 slightly decreased the pH from 6.5 to values ranging from 4 to 5.5, resulting in a moderate increase in biomass (0.5 < OD < 1). Strains ACUF773 and ACUF648 reached the highest biomass yields (1 < OD < 1.5,), as a result of growing acidification of the external medium (pH < 4). Strain ACUF551 decreased the pH to more acidic value, as well, and exhibited the best growth rate, exceeding 1.5 OD after 2 weeks.

From an initial pH 6, all strains were able to lower the pH to similar values (pH < 4), although with growth rates significantly variable among them. ACUF722 and CloneT18 did not exceed OD 1 after 2 weeks, whereas strains ACUF773, ACUF769, and ACUF731 had an OD ranging from 1 to 1.5. The highest biomass yields were reached by ACUF648, ACUF551, and IPPAS P507, with OD > 1.5.

At pH 5, all *G. maxima* strains exceeded OD 2 and lowered pH below 4. Noteworthy was that strains IPPAS 507, ACUF648, ACUF551, and ACUF773 reached the highest biomass yield (1.5 < OD < 2). At this starting pH level, the media conditions were sufficient for the *G. maxima* to alter conditions to maximal growth capacities.

After 6 weeks, all strains, regardless of the initial pH, strongly acidified the external medium (final pH < 3), except IPPAS P507, confirming, therefore, its disability to lower the pH (Figure 3b). However, the differences in growth rate among strains were still more accentuated (0.7 < OD < 18). From an initial pH 6.5, the best growth rate was recorded by ACUF551 (OD = 17.960, Figure 2b), while ACUF722, CloneT18, ACUF773, and ACUF769 had a slower growth rate (OD = 2.955, 4.21, 5.12, 5.465, respectively; Figure 3b). From pH 6, ACUF769 and ACUF722 reached the highest (OD = 14.820) and the lowest (OD = 3.695) biomass yields, respectively; the best growth performances at initial pH 5 were recorded by IPPAS P507 (17.640), while ACUF722 again did not exceed OD 5.

### 3.3. Effect of pH and Nitrogen Source on Growth and Chlorophyll Content

**Figure 4:**
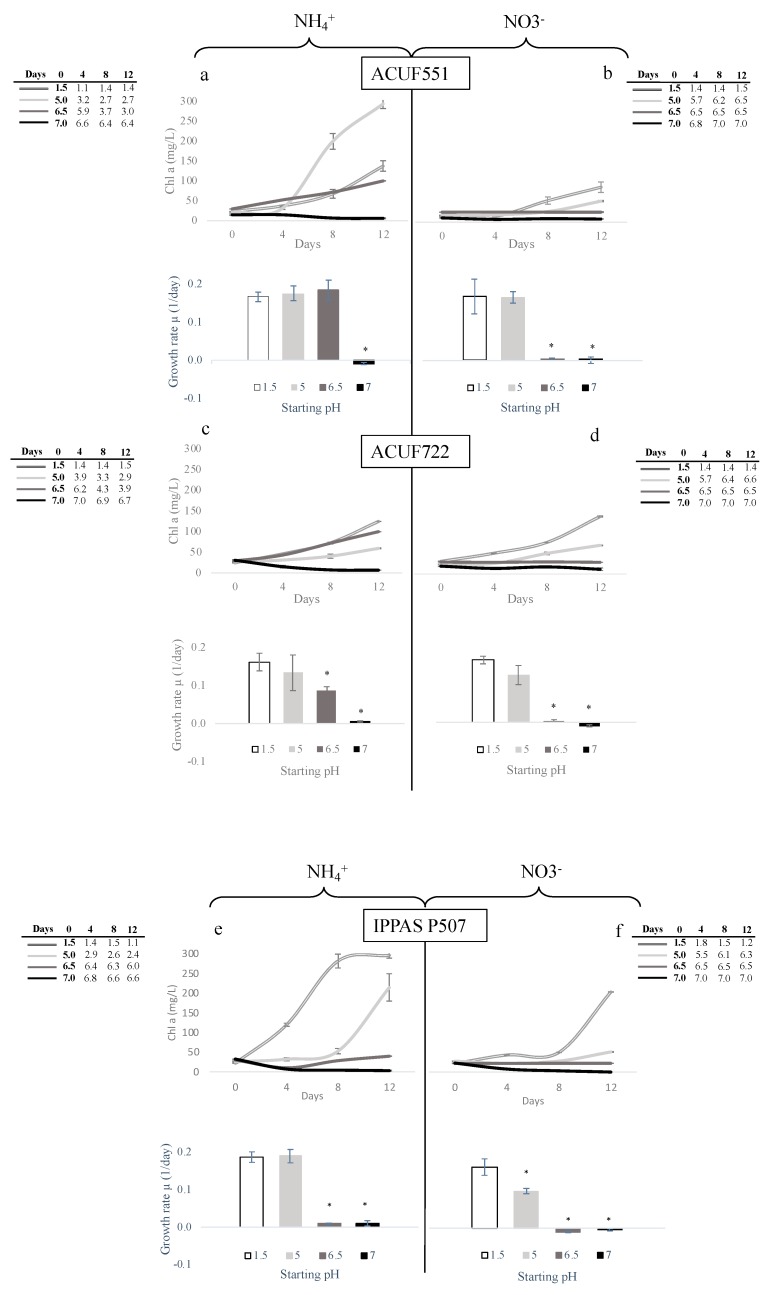
The trend of chlorophyll content for 12 days. The column charts indicate the growth rate measured at the end of the experiments. Data are shown as the mean value (± S.D.), *n* = 3; Values marked with an asterisk are significantly different from the control (Tukey test; *p* < 0.05). Tables on the sides of the graphs represent the pH variations along the experiments.

The combined influence of nitrogen source and pH was tested on 3 *G. maxima* strains displaying different pH responses (Figure 4). We chose ACUF551, whose final biomass yield was comparable to its control, ACUF722, which showed slow growth in all pH conditions, compared to pH 1.5, and IPPAS P507 as the only strain unable to lower the external pH from 6.5.

*G. maxima* ACUF551 grew similarly on either ammonium or nitrate, at pH 1.5, even if the chlorophyll content was significantly higher on NH_4_^+^ than on NO_3_^−^ (Figure 3a,b; 137.15mg/L on NH_4_^+^ *vs* 76.23mg/L on NO_3_^−^; *P* < 0.05). After 2 weeks from an initial pH 5 on ammonium, chlorophyll content was 2.14 fold compared to the control (293.33mg/L; *p* < 0.05); the algal strain did not exhibit a difference in growth rates at pH 5 and 6.5, compared to the control; acidification of the medium progressively occurred, reaching 2.7 and 3.0, respectively (Figure 4a). Interestingly, *G. maxima* ACUF551 showed the ability to grow on nitrate at pH 5, despite the progressive increasing pH of the medium, which reached 6.5 after 2 weeks (Figure 4b). Chlorophyll levels were detected to increase throughout the experiments, despite achieving values lower than in the controls.

**Figure 5:**
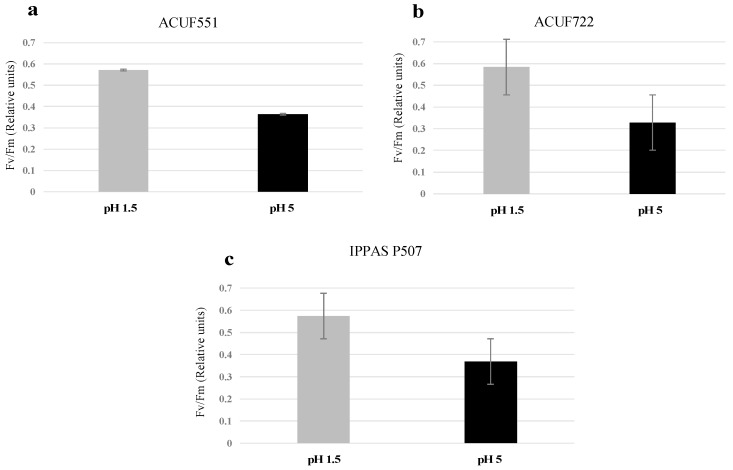
Fv/Fm values of the three *G. maxima* strains (**a**, *G. maxima* ACUF 551; **b**, *G. maxima* ACUF 722; **c**, *G. maxima* IPPAS P507) in the presence of nitrate pH 1.5 and 5; the quantum efficiency of photosystem II (Fv/Fm) was recorded after 12 days. Data are shown as the mean value (± S.D.), *n* = 3; values at pH 5 differed significantly from control (*p* < 0.05).

Comparable growth rates and chlorophyll contents were observed in *G. maxima* ACUF722 on both nitrogen sources at pH 1.5 (Figure 4c,d). A non-significant decrease in growth rate was recorded at pH 5 on ammonium with progressive acidification of the medium (final pH 2.9, Figure 5a), as well as on nitrate, where alkalization took pH at 6.6 (Figure 5b).

As for ACUF551, no significant differences were observed in the IPPAS P507 growth rate, when cultivated at pH 1.5 or pH 5 (final pH 2.4) on ammonium (Figure 4e). From an initial pH 5, chlorophyll levels increased, reaching 214 mg/L after a lag phase in the first 8 days, progressively acidifying the medium. When the medium was supplemented with nitrate instead of ammonia, the progressive increase of pH from 5 up to 6.3 significantly affected both the growth rate and the chlorophyll content (Figure 4f).

Null growth rates were observed for all strains, at pH 6.5 on nitrate and at pH 7 on both N-sources.

### 3.4. Effect of pH and Nitrogen Source on Photochemical Activity

**Figure 6:**
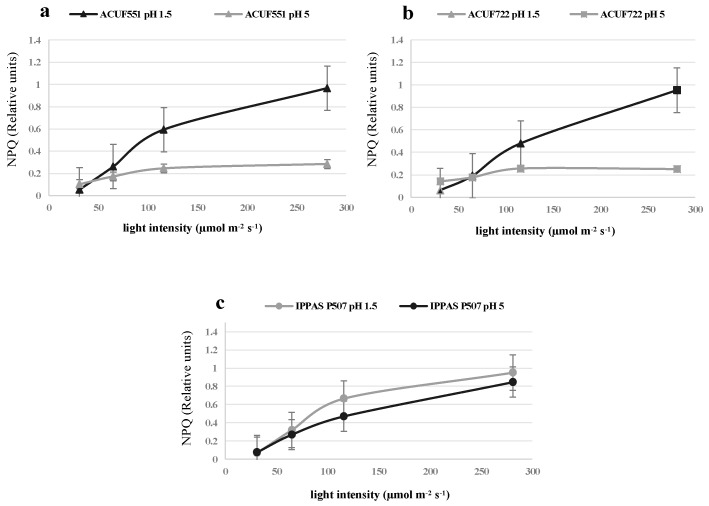
Non-photochemical quenching (NPQ) values measured under nitrate source at pH 1.5 and 5, at different light intensities after 288h (**a**, *G. maxima* ACUF 551; **b**, *G. maxima* ACUF 722; **c**, *G. maxima* IPPAS P507). Data are shown as the mean value (± S.D.), *n* = 3; *G. maxima* ACUF 551 (**a**) and ACUF 722 (**b**) at pH 5 significantly differed from their respective controls (Tukey test; *p* < 0.05).

**Figure 7:**
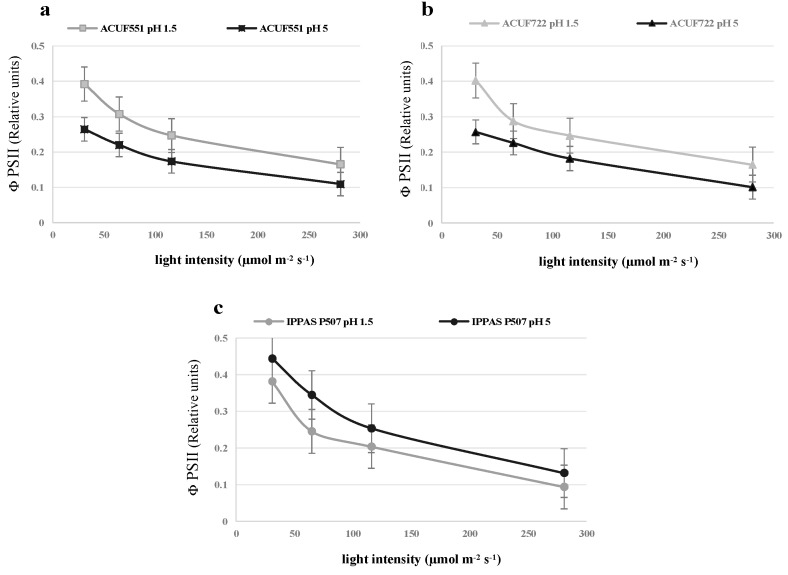
PSII values measured under nitrate source at pH 1.5 and 5, at different light intensities after 288h (**a**, *G. maxima* ACUF 551; **b**, *G. maxima* ACUF 722; **c**, *G. maxim*a IPPAS P507). Data are shown as the mean value (± S.D.), *n* = 3.

Photochemical performances were evaluated by rapid and non-invasive PAM fluorometry. From an initial pH 1.5 with either nitrogen sources, the efficiency of photochemical activity was optimal and comparable (Figure 5, Figure 6 and Figure 7, Figures S1–S9). Under ammonium and nitrate sources, the strains had similar values of Fv/Fm around 0.5–0.6 (values ≥ 0.5, Figure 5a–c). The maximum quantum yield was recorded at 31 μmol photons m^−2^ s^−1^, both on ammonium and on nitrate (0.4, Figure 7a–c, Figures S6–S9), with relatively low NPQ values (0.05–0.08, Figure 6a–c, Figures S2–S5).

Different photochemical performances were noted from a starting pH 5 among the strains on both nitrogen sources: The photochemical efficiency was comparable to pH 1.5, on NH_4_^+^ (Fv/Fm ≥ 0.5, Figure 5a–c; Figure S1), while on nitrate Fv/Fm was significantly lower than ammonium sources (Fv/Fm ≤ 0.4; *p* < 0.05; Figure 5a–c, Figure S1) and only small variations of NPQ and ΦPSII were recorded (Figure 6a–c and Figure 7a–c, Figure S2–S9). 

From pH 6.5, the photochemical activity of ACUF551 and ACUF722 on ammonium was inhibited until 96 hours (Figures S2, S3, S6, S7). After this time as an acclimation period, physiological activity started again (Figures S4, S5, S8, S9). The Russian IPPAS P507 showed an acclimation period of 192 h on ammonium, exhibiting a lower photochemical activity compared to the other strains (Figures S1–S9). No photochemical activity was recorded on nitrate at pH 6.5 in all strains, and neither at pH 7 in all the isolates in the presence of both nitrogen sources. 

## 4. Discussion

We applied different methods to assess the influence of pH and nitrogen source on the physiology of eight *G. maxima* strains. Our results showed a significant correlation between the growth rates and the pH lowering on NH_4_^+^ medium in different strains collected from acidic and non-acidic geothermal environments. It is known that the assimilation of ammonium produces protons, which must be promptly extruded to avoid cell acidification and support metabolism [37]. All strains used in this work showed comparable abilities in lowering the pH when cultured at pH 5 and 6 in nitrogen provided as NH_4_^+^, although with a variable growth rate.

Substantial differences among them were recorded at pH 6.5; the Russian IPPAS P507 required a more extended phase of adaptation, and this strain scarcely grew during the 6 weeks of examination. A decrease in the external pH reached only a value of 5.7. In contrast, the Icelandic ACUF551 and the Turkish ACUF769, both isolated from acidic sites, showed the best growth performances at pH 6.5 and 6, respectively, during the whole experiment. As reported by Lowell and Castenholz (2013), a different ability in lowering pH was also shown by *C. merolae*: Eight strains isolated from Yellowstone were able to reduce the pH of the medium, while none of the three Japanese isolates was competent; the authors also highlighted variations in the final yields, ascribing them both to small differences in inoculum density and in real genetic differences among the competent strains [22]. Among our isolates, two of the Turkish strains (ACUF773, ACUF648) from non-acidic sites quickly lowered the pH to values 3.39-3.42, thus allowing a faster growth, which could be a reliable survival strategy in otherwise hostile environments. According to Reysenbach et al. (2002), in some hot springs, reduced constituents such as hydrogen sulfide, ammonia, and methane are far from equilibrium with their oxidized counterparts such as sulfate, nitrate, and carbon dioxide [38]. Ammonia concentrations strongly vary in these environments, as well as ambient pH. Such ecological variations can produce radically different habitats at a small scale and change rapidly over time as well, selecting for organisms that respond quickly to these geochemical fluctuations.

Another interesting aspect emerging from our experiments is related to the growth in the nitrate-rich medium. All *G. maxima* strains were able to use nitrate as well as ammonium. The dominant nitrogen source in acidic hot springs is ammonium, while nitrate and nitrite are usually generated by the oxidizing activity of the microbial community [38]. As a consequence, nitrate assimilation is rarely essential in these environments [39]. The enzymes usually involved in nitrate assimilation and conversion are nitrate reductase (NR, nitrate to nitrite) and nitrite reductase (NiR, nitrite to ammonium). Among Cyanidiophyceae, *C. merolae* can grow on both nitrogen sources [39]. However, no typical NiR coding gene was found in its complete genome [40]. According to Imamura et al. (2010), the activity is encoded by two candidate genes for sulfite reductase (SiR), located next to the nitrate-related genes. These transcripts are inhibited by ammonium [39]. Similarly, the species *G. sulphuraria* lacks the common NR-coding gene, thus suggesting again the existence of an unusual assimilatory system (Weber unpublished sited in Imamura et al. 2010). By phylogenetic analysis, *G. maxima* is more strictly related to *C. merolae* than to *G. sulphuraria*, despite the striking morphological divergences among the two genera [9,12,13]. It is intriguing that *G*. *maxima* is provided by a nitrate assimilation gene toolkit similar to that of *C. merolae*, thus supporting their phylogenetic relationships (H.S. Yoon, personal communication).

In our pH 1.5 experiments, all cultures were able to use nitrate in a manner significantly comparable to ammonium. These maintained a relatively constant external pH. Photosynthetic efficiency was productive from a low light intensity recorded in all *G. maxima* cultures at pH 1.5 for either nitrate or ammonium, showing Fv/Fm values around 0.500. The maximum efficiency of photosystem II in the dark (Fv/Fm) usually provides indications on the physiological state of the cultures. Red algae exhibit an optimum Fv/Fm that is lower than the ones manifested by green and brown microalgae (0.700). This reduction is related to the presence of phycobiliproteins [11] in the light-harvesting system coupled to PSII, interfering with the fluorescence signal [41]. A steady rise of pH is the result of nitrate uptake and successive transformation to ammonium, requiring a net influx of protons from the culture medium [37]. Cultivation on nitrate from an initial pH 5, and the subsequent increase of the medium pH, correlated with the photochemical activity. Lower values of Fv/Fm, NPQ, and ΦPSII, suggested a stress condition that could influence the protein expression and enzymatic concentration of photosynthetic apparatus [42]. 

Chlorophyll fluorescence represents a valuable bioindicator of the status of the Photosystem II; both Fv/Fm and ΦPSII values are generally employed to express the PSII quantum yield; according to Kalaji et al., [43,44,45] ΦPSII is more suitable than Fv/Fm, since the linear correlation between electron transport rate and CO_2_ assimilation rate has been experimentally demonstrated in several conditions. Contrarily, the authors underline that the parameter Fv/Fm has not been derived experimentally, but it is only based on an analysis of competitive rate equations for the F_0_ and F_M_ states; this analysis is correct only when the fluorescence rise between F_0_ and F_M_ is determined by the reduction of Q_A,_ the primary quinone electron acceptors of PSII [43,44,45].

Fluorometric analyses were used to demonstrate an exponential increase in the growth rate at the beginning of our experiments (0–4 days). These remained constant from the 4th day onward; the survival of the cultures is potentially ascribable to switching from the autotrophic metabolism to a heterotrophic one. Oesterhelt et al. (2007) assessed that autotrophic cultures of *G. sulphuraria* (strain 074) exponentially grew up to pH 5 and reduced the growth significantly at higher pHs. Conversely, heterotrophic cultures of the same strain exhibited cell growth up to pH 8 [46].

Several studies already reported the ability of microorganisms collected from acidic or alkaline environments to survive in a wide range of pH, suggesting these capacities as indicators of the ecological resilience of extremophiles [47]. The discovery of *G. maxima* strains from non-acidic thermal areas and the capacity to tolerate a wide range of initial environmental pHs are strong evidence of their potential adaptation to changing environments. As reported by Lowell and Castenholtz (2013), the ability of several Cyanidiophycean strains in lowering the pH could be an important survival strategy in confined environments, such as microbial mats, interstitial soil spaces, and endolithic niches, rendering them able to survive in non-acidic environments [22]. This could potentially serve as a connection between the thermoacidic locations as a mechanism of long-distance migration [3,20]. According to Dhakar and Pandey (2016), survival in sudden adverse environmental conditions is the consequence of the expression of “hidden” genomic information (Cryptic Genetic Variation, CGV). The changes in ecological factors induce the expression of hidden genes without any alteration in the genetic composition or proper effect on phenotype; transcriptomics and proteomics can be helpful to reveal the change in the cellular response to pH changes [1]. The ability of the extremophiles to respond at environmental variations, such as pH, lies in the expression and production of proteins, extremozymes, or secondary metabolites. These compounds are structurally suitable and of a broad interest in biotechnological processes, including both environmental (bioremediation, biodegradation, and biocontrol), and industrial applications. We see these as sources of new extremozymes and other useful bioactive compounds with wide pH range tolerance.

## Figures and Tables

**Table 1:** Code, name of the collection sites, environmental pH, and temperature.

Strains	Sampling Site	Temperature (°C)	pH
IPPAS_P507	Kunashir (RUS)	41.5 °C	4
ACUF769	Güçlükonak (TURK)	60 °C	1
ACUF722	Güçlükonak (TURK)	60 °C	1
CloneT18	Diyadin (TURK)	45 °C	7
ACUF773	Diyadin (TURK)	45 °C	7
ACUF648	Kula, Manisa (TURK)	41 °C	7
ACUF731	Kula, Manisa (TURK)	41 °C	7
ACUF551	Landmannalaugar, Hekla (ICE)	42 °C	1

## Supplementary Materials

The following are available online at https://www.mdpi.com/2223-7747/9/2/232/s1, Figure S1: Fv/Fm values of the three *G. maxima* strains (a,b *G. maxima* ACUF 551; c,d *G. maxima* ACUF 722; e,f *G. maxima* IPPAS P507) in the presence of ammonium and nitrate under different pHs; the quantum efficiency of photosystem II (Fv/Fm) was recorded at time 0 and after 4, 8, and 12 days. Data are shown as the mean value (± S.D.), *n* = 3; *p* < 0.05. Figure S2: NPQ values measured under ammonium and nitrate source at different light intensities at the beginning of the experiment (a,b *G. maxima* ACUF 551; c,d *G. maxima* ACUF 722; e,f *G. maxima* IPPAS P507). Figure S3: NPQ values measured under ammonium and nitrate source at different light intensities after 4 days (a,b *G. maxima* ACUF 551; c,d *G. maxima* ACUF 722; e,f *G*. *maxima* IPPAS P507). Figure S4: NPQ values measured under ammonium and nitrate source at different light intensities after 8 days (a,b *G. maxima* ACUF 551; c,d *G. maxima* ACUF 722; e,f *G. maxima* IPPAS P507). Figure S5: NPQ values measured under ammonium and nitrate source under different pHs, at different light intensities after 12 days (a,b *G. maxima* ACUF 551; c,d *G. maxima* ACUF 722; e,f *G. maxima* IPPAS P507). Figure S6: PSII values measured under ammonium and nitrate source at different light intensities at the beginning of the experiment (a,b *G. maxima* ACUF 551; c,d *G. maxima* ACUF 722; e,f *G. maxima* IPPAS P507). Figure S7: ΦPSII values measured under ammonium and nitrate source at different light intensities after 4 days (a,b *G. maxima* ACUF 551; c,d *G. maxima* ACUF 722; e,f *G. maxima* IPPAS P507). Figure S8: ΦPSII values measured under ammonium and nitrate source at different light intensities after 8 days (a,b *G. maxima* ACUF 551; c,d *G. maxima* ACUF 722; e,f *G. maxima* IPPAS P507). Figure S9: ΦPSII values measured under ammonium and nitrate source under different pHs, at different light intensities after 12 days (a,b *G. maxima* ACUF 551; c,d *G. maxima* ACUF 722; e,f *G. maxima* IPPAS P507). Table S1: Taxa, collection site, strain voucher, and accession numbers (*rbcL*) for the dataset. Table S2: Optical densities and pHs measured weekly during six weeks of growth under ammonium as the nitrogen source starting from pH 7, 6.5, 6, 5, and 1.5. Table S3: Optical densities, chlorophyll content, and pHs measured during 12 Days of growth under ammonium or nitrate as the nitrogen source starting from pH 7, 6.5, 5, and 1.5.

## References

[1] Dhakar K., Pandey A. (2016). Wide pH range tolerance in extremophiles: Towards understanding an important phenomenon for future biotechnology. Appl. Microbiol. Biotechnol..

[2] Seckbach J., Oren A., Stan-Lotter H. (2013). Polyextremophiles: Life under Multiple Forms of Stress.

[3] Brock T.D. (1978). The genus Cyanidium. Thermophilic Microorganisms and Life at High Temperatures.

[4] Albertano P., Pinto G., Pollio A. (1994). Ecophysiology and Ultrastructure of an Acidophilic Species of Ochromonas (Chrysophyceae, Ochromonadales). Arch. für Protistenkd..

[5] Schönknecht G., Chen W., Ternes C.M., Barbier G.G., Shrestha R.P., Stanke M., Bräutigam A., Baker B.J., Banfield J.F., Garavito R.M. (2013). Gene Transfer from Bacteria and Archaea Facilitated Evolution of an Extremophilic Eukaryote. Science.

[6] Ciniglia C., Yoon H.S., Pollio A., Pinto G., Bhattacharya D. (2004). Hidden biodiversity of the extremophilic Cyanidiales red algae. Mol. Ecol..

[7] Toplin J.A., Norris T.B., Lehr C.R., McDermott T.R., Castenholz R.W. (2008). Biogeographic and phylogenetic diversity of thermoacidophilic cyanidiales in Yellowstone National Park, Japan, and New Zealand. Appl. Environ. Microbiol..

[8] Skorupa D.J., Reeb V., Castenholz R.W., Bhattacharya D., Mcdermott T.R. (2013). Cyanidiales diversity in Yellowstone National Park. Lett. Appl. Microbiol..

[9] Ciniglia C., Yang E.C., Pollio A., Pinto G., Iovinella M., Vitale L., Yoon H.S. (2014). Cyanidiophyceae in Iceland: Plastid rbc L gene elucidates origin and dispersal of extremophilic Galdieria sulphuraria and G. maxima (Galdieriaceae, Rhodophyta). Phycologia.

[10] Hsieh C.J., Zhan S.H., Lin Y., Tang S.L., Liu S.L. (2015). Analysis of rbcL sequences reveals the global biodiversity, community structure, and biogeographical pattern of thermoacidophilic red algae (Cyanidiales). J. Phycol..

[11] Carfagna S., Landi V., Coraggio F., Salbitani G., Vona V., Pinto G., Pollio A., Ciniglia C. (2018). Different characteristics of C-phycocyanin (C-PC) in two strains of the extremophilic Galdieria phlegrea. Algal Res..

[12] Eren A., Iovinella M., Yoon H.S., Cennamo P., de Stefano M., de Castro O., Ciniglia C. (2018). Genetic structure of Galdieria populations from Iceland. Polar Biol..

[13] Iovinella M., Eren A., Pinto G., Pollio A., Davis S.J., Cennamo P., Ciniglia C. (2018). Cryptic dispersal of Cyanidiophytina (Rhodophyta) in non-acidic environments from Turkey. Extremophiles.

[14] Del Mondo A., Iovinella M., Petriccione M., Nunziata A., Davis S.J., Cioppa D., Ciniglia C. (2019). A SPOTLIGHT ON RAD52 IN CYANIDIOPHYTINA (RHODOPHYTA): A RELIC IN ALGAL HERITAGE. Plants.

[15] Carfagna S., Bottone C., Cataletto P.R., Petriccione M., Pinto G., Salbitani G., Vona V., Pollio A., Ciniglia C. (2016). Impact of sulfur starvation in autotrophic and heterotrophic cultures of the Extremophilic Microalga Galdieria Phlegrea (Cyanidiophyceae). Plant Cell Physiol..

[16] Sentsova Y.O. (1991). On the diversity of acido-thermophilic unicellular algae of the genus Galdieria (Rhodophyta, Cyanidiophyceae). Bot. J..

[17] Rigano C., Fuggi A., Rigano V.D.M., Aliotta G. (1976). Studies on utilization of 2-ketoglutarate, glutamate and other amino acids by the unicellular alga Cyanidium caldarium. Arch. Microbiol..

[18] Rigano C., Aliotta G., Rigano Martino Di V., Fuggi A., Vona V. (1977). Heterotrophic Growth Patterns in the Unicellular Alga Cyanidium Calderium. Arch. Microbiol..

[19] Gross W., Schnarrenberger C. (1995). Heterotrophic Growth of Two Strains of the Acido-Thermophilic Red Alga Galdieria sulphuraria. Plant Cell Physiol..

[20] Gross W., Oesterhelt C. (1999). Ecophysiological studies on the red alga Galdieria sulphuraria isolated from southwest Iceland. Plant Biol..

[21] Oesterhelt C., Schnarrenberger C., Gross W. (1999). Characterization of a sugar/polyol uptake system in the red alga Galdieria sulphuraria. Eur. J. Phycol..

[22] Lowell C., Castenholz R.W. (2013). The lowering of external pH in confined environments by thermo-acidophilic algae (class: Cyanidiophyceae). Environ. Microbiol. Rep..

[23] Henkanatte-Gedera S.M., Selvaratnam T., Karbakhshravari M., Myint M., Nirmalakhandan N., Van Voorhies W., Lammers P.J. (2017). Removal of dissolved organic carbon and nutrients from urban wastewaters by Galdieria sulphuraria: Laboratory to field scale demonstration. Algal Res..

[24] Wang J., Curtis W.R. (2016). Proton stoichiometric imbalance during algae photosynthetic growth on various nitrogen sources: Toward metabolic pH control. J. Appl. Phycol..

[25] Allen M.M., Stanier R.Y. (1968). Selective Isolation of Blue-green Algae from Water and Soil. J. Gen. Microbiol..

[26] Ciniglia C., Mastrobuoni F., Scortichini M., Petriccione M. (2015). Oxidative damage and cell-programmed death induced in Zea mays L. by allelochemical stress. Ecotoxicology.

[27] Edgar R.C. (2004). MUSCLE: Multiple sequence alignment with high accuracy and high throughput. Nucleic Acids Res..

[28] Okonechnikov K., Golosova O., Fursov M. (2012). Unipro UGENE: A unified bioinformatics toolkit. Bioinformatics.

[29] Darriba D., Taboada G.L., Doallo R., Posada D. (2012). jModelTest 2: More models, new heuristics and parallel computing. Nat. Methods.

[30] Stamatakis A. (2014). RAxML version 8: A tool for phylogenetic analysis and post-analysis of large phylogenies. Bioinf. Appl..

[31] Juneau P., Green B.R., Harrison P.J. (2005). Simulation of Pulse-Amplitude-Modulated (PAM) fluorescence: Limitations of some PAM-parameters in studying environmental stress effects. Photosynthetica.

[32] Hanelt D., Nultsch W. (1995). Field Studies of Photoinhibition Show Non-Correlationsbetween Oxygen and Fluorescence Measurements in the Arctic Red Alga Palmaria palmata. J. Plant Physiol..

[33] Carbone D.A., Gargano I., Pinto G., De Natale A., Pollio A. (2017). Evaluating Microalgae Attachment to Surfaces: A first Approach towards a Laboratory Integrated Assessment. Chem. Eng. Trans..

[34] Carbone D.A., Gargano I., Olivieri G., Marzocchella A., Andreozzi R., Marotta R., Spasiano D., Pinto G., Pollio A. (2019). LIGHT INTENSITIES MAXIMIZING PHOTOSYNTHESIS AND KINETICS OF PHOTOCHEMICAL STEPS IN Graesiella emersonii UNDER DIFFERENT CULTIVATION STRATEGIES. Environ. Eng. Manag. J..

[35] Schreiber U., Schliwa U., Bilger W. (1986). Continuous recording of photochemical and non-photochemical chlorophyll fluorescence quenching with a new type of modulation fluorometer. Photosynth. Res..

[36] R Core Team (2014). R: A Language and Environment for Statistical Computing.

[37] Scherholz M.L., Curtis W.R. (2013). Achieving pH control in microalgal cultures through fed-batch addition of stoichiometrically-balanced growth media. BMC Biotechnol..

[38] Reysenbach A.-L., Shock E. (2002). Merging Genomes with Geochemistry in Hydrothermal Ecosystems. Science.

[39] Imamura S., Terashita M., Ohnuma M., Maruyama S., Minoda A., Weber A.P.M., Inouye T., Sekine Y., Fujita Y., Omata T. (2010). Nitrate assimilatory genes and their transcriptional regulation in a unicellular red alga cyanidioschyzon merolae: Genetic evidence for nitrite reduction by a sulfite reductase-like enzyme. Plant Cell Physiol..

[40] Nozaki H., Takano H., Misumi O., Terasawa K., Matsuzaki M., Maruyama S., Nishida K., Yagisawa F., Yoshida Y., Fujiwara T. (2007). A 100%-complete sequence reveals unusually simple genomic features in the hot-spring red alga Cyanidioschyzon merolae. BMC Biol..

[41] Büchel C., Wilhelm C. (1993). In vivo analysis of slow chlorophyll fluorescence induction kinetics in algae: Progress, problems and perspectives. Photochem. Photobiol..

[42] Salleh S., McMinn A. (2011). THE EFFECTS OF TEMPERATURE ON THE PHOTOSYNTHETIC PARAMETERS AND RECOVERY OF TWO TEMPERATE BENTHIC MICROALGAE, AMPHORA CF. COFFEAEFORMIS AND COCCONEIS CF. SUBLITTORALIS (BACILLARIOPHYCEAE)1. J. Phycol..

[43] Kalaji H.M., Schansker G., Brestic M., Bussotti F., Calatayud A., Ferroni L., Goltsev V., Guidi L., Jajoo A., Li P. (2017). Frequently Asked Questions about Chlorophyll Fluorescence, the Sequel.

[44] Kalaji H.M., Schansker G., Ladle R.J., Goltsev V., Bosa K., Allakhverdiev S.I., Brestic M., Bussotti F., Calatayud A., Dąbrowski P. (2014). Frequently asked questions about in vivo chlorophyll fluorescence: Practical issues. Photosynth. Res..

[45] Kalaji H.M., Jajoo A., Oukarroum A., Brestic M., Zivcak M., Samborska I.A., Cetner M.D., Łukasik I., Goltsev V., Ladle R.J. (2016). Chlorophyll a fluorescence as a tool to monitor physiological status of plants under abiotic stress conditions. Acta Physiol. Plant..

[46] Oesterhelt C., Schmälzlin E., Schmitt J.M., Lokstein H. (2007). Regulation of photosynthesis in the unicellular acidophilic red alga Galdieria sulphuraria†. Plant J..

[47] Pandey A., Dhakar K., Sharma A., Priti P., Sati P., Kumar B. (2015). Thermophilic bacteria that tolerate a wide temperature and pH range colonize the Soldhar (95 °C) and Ringigad (80 °C) hot springs of Uttarakhand, India. Ann. Microbiol..

