# Supplementary material for: Prevalent pH Controls the Capacity of Galdieria maxima to Use Ammonia and Nitrate as a Nitrogen Source
Source: Plants (Basel). 2020 Feb 11;9(2):232. doi: 10.3390/plants9020232 (PMC7076501; doi:10.3390/plants9020232)
Supplement: Supplementary file 1 [file plants-09-00232-s001.zip › Supplementary materials/TabS1.pdf]

**Taxa**	**Collection site**	**Strain Voucher**	**Accession Number**
Galdieria	Kunashir (Russia)	IPPAS P507	AY391370
maxima	Landmannalaugar (Iceland)	ACUF551	MK394995
		ACUF419	KC883840
		ACUF420	KC883841
		ACUF421	KC883842
		ACUF428	KC883848
		ACUF449	KC883861
		ACUF450	KC883862
		ACUF456	KC883868
		ACUF457	KC883869
		ACUF458	KC883870
		ACUF451	KC883863
	Seltun (Iceland)	ACUF389	KC883816
		ACUF393	KC883819
		ACUF396	KC883821
		ACUF425	KC883846
		ACUF436	KC883849
		ACUF468	KC883880
		ACUF469	KC883881
		ACUF411	KC883833
	Niasjvellir (Iceland)	ACUF404	KC883827
	Güçlükonak (Turkey)	ACUF769	KX501179
		ACUF722	KX501174
		ACUF660	KY033404
		ACUF710	KX501173
		ACUF697	KY033415
	Diyadin (Turkey)	cloneT18	KX501185
		ACUF773	KX501180
		ACUF665	KY033406
		ACUF774	KY033436
		cloneT06	KY033453
		ACUF772	KY033435
	Manisa Kula (Turkey)	ACUF648	KY033397
		ACUF731	KY033420
		ACUF671	KY033410
		ACUF743	KY033428
		ACUF777	KY033439
		ACUF776	KY033438
	Germencik (Turkey)	cloneT15	KX501183
		ACUF673	KY033411
		ACUF739	KY033425
		ACUF742	KY033427
	Cermick (Turkey)	ACUF647	KY033396
		cloneT03	KY033450

		cloneT04	KY033451
		ACUF650	KY033398
		ACUF766	KY033431
		ACUF783	KY033443
	Biloris (Turkey)	ACUF653	KY033400
		ACUF763	KY033429
		ACUF764	KY033430
		ACUF735	KY033422
Galdieria sulphuraria	Yellostone National Park (USA)	SAG108.79	AY119767
	California (USA)	UTEX2393	AF233069
	Caserta (ITaly)	ACUF011	AY541303
	Los Azufres (Mexico)	ACUF135	AY541309
	Benevento (Italy)	ACUF012	AY541310
	Solfatara (Italy)	ACUF017	AY541306
	Scarfoglio (Italy)	ACUF018	AY541304
	Vulcano (Italy)	ACUF021	AY541307
	Ischia (Italy)	ACUF015	AY541305
	Sasso Pisano (Italy)	SP3-C2	DQ916749
	Sasso Pisano (Italy)	SP1-10	DQ916748
	Monte Rotondo (Italy)	MR6-C36	DQ916747
		MR5-C17	DQ916746
		MR4-21	DQ916745
	Pisciarelli (Italy)	cloneA12	AY541313
		cloneD5	AY541321
		cloneD15	AY541322
		cloneE11	AY541324
		cloneE12	AY541325
	Gunnhuver (Iceland)	ACUF381	KC883808
		ACUF382	KC883809
	Landmannalaugar (Iceland)	ACUF385	KC883812
		ACUF386	KC883813
		ACUF387	KC883814
		ACUF388	KC883815
	Seltun (Iceland)	ACUF395	KC883820
		ACUF397	KC883822
		ACUF398	KC883973
		ACUF422	KC883843
		ACUF423	KC883844
		ACUF424	KC883845
		ACUF437	KC883850
		ACUF409	KC883831
		ACUF448	KC883860
		ACUF452	KC883864
		ACUF439	KC883852
		ACUF454	KC883866

		ACUF440	KC883853
		ACUF470	KC883882
		ACUF472	KC883883
		ACUF473	KC883884
		ACUF474	KC883885
		ACUF475	KC883886
		ACUF459	KC883871
		ACUF460	KC883872
		ACUF410	KC883832
		ACUF463	KC883875
		ACUF417	KC883839
		ACUF412	KC883834
	Niasjvellir (Iceland)	ACUF399	KC883823
		ACUF400	KC883824
		ACUF443	KC883855
		ACUF444	KC883856
	Viti (Iceland)	ACUF461	KC883873
	Güçlükonak (Turkey)	ACUF658	KY033403
		ACUF725	KX501175
		ACUF768	KX501178
		ACUF781	KY033441
	Germencik (Turkey)	ACUF778	KX501181
		ACUF674	KY033412
		ACUF676	KY033413
		ACUF779	KY033440
		cloneT02	KY033449
Galdieria partita	Kamchatka (Russia)	IPPAS P500	AB18008
Galdieria daedala	Kunashir (Russia)	IPPAS P508	AY541302
Galdieria phlegrea	Viterbo (Italy)	ACUF009	AY119768
	Agrigento (Italy)	ACUF063	AY119769
	Pisciarelli (Italy)	ACUF002	AY541311
		cloneB15	AY541314
		cloneB19	AY541315
		cloneB20	AY541316
		cloneC1	AY541317
	Biloris (Turkey)	ACUF765	KX501177
		ACUF652	KY033399
		ACUF657	KY033402
		ACUF780	KX501182
		ACUF656	KY033401
	Güçlükonak (Turkey)	ACUF784	KY033444
		cloneT09	KY033456

		cloneT16	KX501184
	Nemrut (Turkey)	ACUF738	KX501176
		ACUF664	KY033405
	Diyadin (Turkey)	ACUF667	KY033407
		ACUF669	KY033409
		ACUF788	KY033447
		ACUF771	KY033434
	Cermik (Turkey)	ACUF642	KY033395
		ACUF625	KY033394
		cloneT07	KY033454
		cloneT08	KY033455
		cloneT10	KY033457
		ACUF668	KY033408
Cyanidioschyzon merolae	Java (Indonesia)	ACUF201	AY119765
	Monte Nuovo (Italy)	ACUF202	AY541296
	Pisciarelli (Italy)	ACUF001	AY119766
		cloneA1	AY541312
		cloneD1	AY541320
		cloneE10	AY541323
	Biloris (Turkey)	cloneT01	KY033448
	Nemrut (Turkey)	cloneT05	KY033452
Cyanidium caldarium	Siena (Italy)	ACUF019	AY541297
	Java (Indonesia)	ACUF182	AY541298
	Acqua Santa (Italy)	ACUF020	AY541299
	Monte Rotondo (Italy)	MR4-22	DQ916750
		MR5-5	DQ916751
		MR6-C35	DQ916752
	Sasso Pisano (Italy)	SP1-10	DQ916753
	Pisciarelli (Italy)	cloneC2	AY541318
	Cermik (Turkey)	ACUF767	KY033432
	Diyadin (Turkey)	ACUF775	KY033437
	Güçlükonak (Turkey)	cloneT17	KY033462
Cyanidium chilense	Monte Rotaro (Italy)	sp.19	AY541300
		sp.20	AY541301
	Terme di baia (Italy)	sp.21	KC914876
Cyanidales sp.	Yellowstone National Park (USA)	DS1-9	JQ269631
		DS2-5	JQ269629
		DS3-1	JQ269633
		SFFL-5	JQ269630
		LCATERR-7	JQ269608
		LCBCEL-5	JQ269609
		LCBTERR-6	JQ269612
		CHJ-4	JQ269635
		CHJ-5	JQ269634
		DSB-9	JQ269617

		DSC-8	JQ269618
		DSD-7	JQ269605
		DSE-8	JQ269619
		DSF-12	JQ269620
		DSH-4	JQ269606
		DS1-6	JQ269623
		DS2-2	JQ269638
		DS3-3	JQ269624
		SFFL-8	JQ269630
		SFFR-7	JQ269616
		LCASUB-11	JQ269627
		LCBCEL-7	JQ269610
		LCBSUB-5	JQ269628
		LCBTERR-12	JQ269611
		LCCBLGR-8	JQ269613
		LCCYEGR-4	JQ269614
		NCB-4	JQ269621
		RIVER1B-5	JQ269636
		SSI-6	JQ269622
		SSII-1	JQ269626
		TS-4	JQ269637
**Outgroups**			
Rhodella violacea	/	SAG115.79	AY119776
Bangiopsis subsimplex	/	PR21	AY119772
Dixoniella grisea	/	SAG39.94	AY119773
Porphyridium aerugineum	/	SAG1380-2	AY119775
