# Supplementary material for: Prevalent pH Controls the Capacity of Galdieria maxima to Use Ammonia and Nitrate as a Nitrogen Source
Source: Plants (Basel). 2020 Feb 11;9(2):232. doi: 10.3390/plants9020232 (PMC7076501; doi:10.3390/plants9020232)
Supplement: Supplementary file 1 [file plants-09-00232-s001.zip › Supplementary materials/TabS3.pdf]

Ammonium					
**Start pH 7**		**Time 0**	**Day 4**	**Day 8**	**Day 12**
ACUF551	OD	0.259 ± 0.006	0.318 ± 0.007	0.275 ± 0.028	0.234 ± 0.029
	Chl	15.123 ± 1.969	14.940 ± 1.839	6.770 ± 1.900	6.087 ± 1.132
	pH	6.9 ± 0.05	7.0 ± 0.02	7.0 ± 0.04	6.9 ± 0.03
ACUF722	OD	0.271 ± 0.013	0.289 ± 0.018	0.283 ± 0.059	0.288 ± 0.016
	Chl	30.867 ± 1.273	15.333 ± 3.055	7.370 ± 1.735	6.680 ± 0.10
	pH	7.00 ± 0.01	7.1 ± 0.04	6.9 ± 0.06	6.7 ± 0.02
IPPAS P507	OD	0.225 ± 0.007	0.240 ± 0.006	0.232 ± 0.059	0.255 ± 0.072
	Chl	32.190 ± 0.375	6.700 ± 1.873	4.149 ± 0.411	2.877 ± 1.051
	pH	7.0 ± 0.05	7.0 ± 0.03	7.04 ± 0.04	7.0 ± 0.01
**Start pH 6.5**		**Time 0**	**Day 4**	**Day 8**	**Day 12**
ACUF551	OD	0.319 ± 0.004	0.726 ± 0.008	1.171 ± 0.008	2.883 ± 0.272
	Chl	29.233 ± 0.493	52.167 ± 0.907	72.033 ± 1.050	100.033 ± 0.208
	pH	6.4 ±0.00	5.9 ± 0.058	3.7 ± 0.06	3.0 ± 0.06
ACUF722	OD	0.338 ± 0.001	0.487 ± 0.007	0.533 ± 0.007	0.960 ± 0.025
	Chl	29.233 ± 0.493	43.433 ± 0.666	73.320 ± 2.282	100.033 ± 0.208
	pH	6.5 ±0.06	6.2 ± 0.02	4.3 ± 0.01	3.9 ± 0.06
IPPAS P507	OD	0.314 ± 0.004	0.300 ± 0.004	0.298 ± 0.003	0.279 ± 0.015
	Chl	29.867 ± 0.058	29.100 ± 0.100	28.500 ± 0.781	27.200 ± 0.721
	pH	6.50 ± 0.06	6.4 ± 0.01	6.3 ± 0.01	6.0 ± 0.01
**Start pH 5**		**Time 0**	**Day 4**	**Day 8**	**Day 12**
ACUF551	OD	0.373 ± 0.20	1.233 ± 0.058	2.437 ± 0.259	3.080 ± 0.035
	Chl	21.967 ± 0.636	31.300 ± 2.941	199.333 ± 19.655	293.333 ± 11.547
	pH	4.9 ± 0.14	3.2 ± 0.02	2.7 ± 0.03	2.7 ± 0.04
ACUF722	OD	0.289 ± 0.023	0.484 ± 0.020	0.756 ± 0.026	1.433 ± 0.104
	Chl	26.930 ± 0.324	31.313 ± 1.242	40.763 ± 5.033	59.620 ± 1.000
	pH	5.2 ± 0.09	3.9 ± 0.03	3.3 ± 0.01	2.9 ± 0.02

IPPAS P507	OD	0.324 ± 0.010	0.590 ± 0.025	1.110 ± 0.108	3.147 ± 0.184
	Chl	25.330 ± 0.754	31.900 ± 4.046	52.350 ± 6.800	227.333 ± 23.029
	pH	4.9 ± 0.05	2.8 ± 0.11	2.6 ± 0.14	2.4 ± 0.09

Start pH 1.5		Time 0	Day 4	Day 8	Day 12
ACUF551	OD	0.414 ± 0.009	0.722 ± 0.030	1.481 ± 0.054	3.044 ± 0.128
	Chl	23.447 ± 0.035	37.370 ± 1.768	70.927 ± 3.677	137.146 ± 13.152
	pH	1.3 ± 0.05	1.05 ± 0.02	1.4 ± 0.06	1.4 ± 0.03
ACUF722	OD	0.396 ± 0.014	0.676 ± 0.004	1.485 ± 0.037	2.743 ± 0.197
	Chl	24.593 ± 0.028	46.353 ± 6.958	72.893 ± 6.081	124.733 ± 4.780
	pH	1.4 ± 0.04	1.4 ± 0.02	1.4 ± 0.02	1.5 ± 0.01
IPPAS P507	OD	0.389 ± 0.009	1.167 ± 0.029	2.237 ± 0.076	3.667 ± 0.153
	Chl	21.700 ± 0.428	119.333 ± 4.041	280.917 ± 17.522	295.333 ± 7.024
	pH	1.1 ± 0.05	1.4 ± 0.02	1.5 ± 0.08	1.1 ± 0.12

Nitrate

Start pH 7		Time 0	Day 4	Day 8	Day 12
ACUF551	OD	0.284 ± 0.008	0.281 ± 0.019	0.308 ± 0.028	0.288 ± 0.003
	Chl	9.534 ± 0.048	6.603 ± 0.068	7.880 ± 0.455	7.407 ± 0.251
	pH	7.0 ± 0.02	6.8 ± 0.01	7.0 ± 0.02	7.0 ± 0.01
ACUF722	OD	0.272 ± 0.008	0.274 ± 0.006	0.300 ± 0.005	0.243 ± 0.024
	Chl	14.643 ± 0.224	10.467 ± 0.301	13.333 ± 0.577	8.967 ± 2.442
	pH	7.0 ± 0.01	6.8 ± 0.04	7.0 ± 0.01	7.0 ± 0.01
IPPAS P507	OD	0.271 ± 0.011	0,226 ± 0.019	0,265 ± 0.038	0.259 ± 0.029
	Chl	22.803 ± 0.267	7.453 ± 0.597	3.267 ± 0.011	0.119 ± 0.009
	pH	7.01 ± 0.03	7.0 ± 0.04	7.0 ± 0.03	7.0 ± 0.03

Start pH 6.5		Time 0	Day 4	Day 8	Day 12
ACUF551	OD	0.307 ± 0.004	0.312 ± 0.004	0.336 ± 0.004	0.330 ± 0.001
	Chl	22-067 ± 0.115	22.467 ± 0.058	22.367 ± 0.252	22.200 ± 0.755

	pH	6.5 ± 0.01	6.5 ± 0.01	6.5 ± 0.01	6.5 ± 0.01
ACUF722	OD	0.315 ± 0.001	0.326 ± 0.005	0.313 ± 0.003	0.329 ± 0.004
	Chl	22.467 ± 0.058	22.067 ± 0.058	22.667 ± 0.058	21.967 ± 0.058
	pH	6.5 ± 0.01	6.5 ± 0.01	6.5 ± 0.01	6.5 ± 0.01
IPPAS P507	OD	0.314 ± 0.004	0.300 ± 0.004	0.298 ± 0.003	0.279 ± 0.015
	Chl	0.334 ± 0.004	0.353 ± 0.005	0.353 ± 0.001	0.329 ± 0.003
	pH	6.50 ± 0.01	6.5 ± 0.01	6.5 ± 0.01	6.5 ± 0.00
**Start pH 5**		**Time 0**	**Day 4**	**Day 8**	**Day 12**
ACUF551	OD	0.250 ± 0.021	0.521 ± 0.020	1.123 ± 0.025	1.767 ± 0.153
	Chl	14.953 ± 0.793	16.313 ± 0.397	23.439 ± 1.607	46.038 ± 1.299
	pH	5.0 ± 0.07	5.7 ± 0.03	6.2 ± 0.05	6.4 ± 0.05
ACUF722	OD	0.269 ± 0.008	0.405 ± 0.024	0.807 ± 0.144	1.181 ± 0.192
	Chl	18.577 ± 0.106	19.887 ± 0.698	38.640 ± 2.359	46.038 ± 1.299
	pH	5.3 ± 0.02	5.7 ± 0.02	6.4 ± 0.05	6.6 ± 0.06
IPPAS P507	OD	0.353 ± 0.013	0.444 ± 0.020	0.790 ± 0.085	1.123 ± 0.068
	Chl	27.747 ± 0.525	22.783 ± 1.788	27.967 ± 1.124	51.333 ± 1.155
	pH	5.1 ± 0.05	5.5 ± 0.09	6.1 ± 0.05	6.3 ± 0.06
**Start pH 1.5**		**Time 0**	**Day 4**	**Day 8**	**Day 12**
ACUF551	OD	0.348 ± 0.021	0.663 ± 0.30	1.509 ± 0.145	2.525 ± 0.464
	Chl	12.667 ± 0.015	13.009 ± 0.429	47.153 ± 7.902	108.167 ± 1.060
	pH	1.5 ± 0.00	1.4 ± 0.00	1.4 ± 0.01	1.5 ± 0.00
ACUF722	OD	0.388 ± 0.008	0.920 ± 0.012	1.296 ± 0.085	2.722 ± 0.298
	Chl	23.730 ± 0.352	39.047 ± 1.001	59.533 ± 0.170	108.167 ± 1.060
	pH	1.5 ± 0.03	1.4 ± 0.03	1.4 ± 0.01	1.4 ± 0.04
IPPAS P507	OD	0.389 ± 0.009	0.829 ± 0.019	1.270 ± 0.073	2.596 ± 0.222
	Chl	21.700 ± 0.428	43.627 ± 4.816	50.053 ± 1.616	204.067 ± 31.126
	pH	1.1 ± 0.05	1.8 ± 0.05	1.4 ± 0.06	1.2 ± 0.02
