# Supplementary material for: Prevalent pH Controls the Capacity of Galdieria maxima to Use Ammonia and Nitrate as a Nitrogen Source
Source: Plants (Basel). 2020 Feb 11;9(2):232. doi: 10.3390/plants9020232 (PMC7076501; doi:10.3390/plants9020232)
Supplement: Supplementary file 1 [file plants-09-00232-s001.zip › Supplementary materials/TabS2.pdf]

| Start pH 7 | | Time 0 | Week 1 | Week 2 | Week 3 | Week 4 | Week 5 | Week 6 |
|---|---|---|---|---|---|---|---|---|
| IPPAS P507 | OD | 0.343 ± 0.006 | 0,.364 ± 0.001 | 0.353 ± 0.001 | 0.312 ± 0.002 | 0.313± 0.005 | 0.317± 0.001 | 0.372 ± 0.004 |
| | pH | 7.01 ± 0.01 | 6.99 ± 0.01 | 7.00 ± 0.01 | 7.02 ± 0.02 | 6.98 ± 0.01 | 7.00 ± 0.01 | 7.01 ± 0.02 |
| ACUF769 | OD | 0.335 ± 0.006 | 0.277 ± 0.002 | 0.317 ± 0.001 | 0.313 ± 0.005 | 0.360 ± 0.018 | 0.313 ± 0.005 | 0.310 ± 0.001 |
| | pH | 6.98 ± 0.01 | 7.00 ± 0.03 | 6.96 ± 0.01 | 7.10 ± 0.14 | 6.96 ± 0.01 | 6.93 ± 0.00 | 6.98 ± 0.01 |
| ACUF722 | OD | 0.310 ± 0.001 | 0.257 ± 0.054 | 0.313 ± 0.004 | 0.321 ± 0.005 | 0.326 ± 0.002 | 0.325 ± 0.001 | 0.324 ± 0.011 |
| | pH | 7.01 ± 0.01 | 6.95 ± 0.00 | 7.01 ± 0.01 | 6.85 ± 0.07 | 6.95 ± 0.02 | 6.95 ± 0.00 | 6.97 ± 0.01 |
| CloneT18 | OD | 0.326 ± 0.002 | 0.296 ± 0.029 | 0.324 ± 0.001 | 0.355 ± 0.011 | 0.348 ± 0.001 | 0.335 ± 0.006 | 0.332 ± 0.001 |
| | pH | 7.01 ± 0.01 | 6.98 ± 0.01 | 6.84 ± 0.08 | 7.10 ± 0.00 | 6.95 ± 0.02 | 6.98 ± 0.01 | 6.97 ± 0.00 |
| ACUF773 | OD | 0.335 ± 0.006 | 0.340 ± 0.001 | 0.306 ± 0.005 | 0.343 ± 0.006 | 0.303 ± 0.001 | 0.343 ± 0.008 | 0.346 ± 0.001 |
| | pH | 7.01 ± 0.00 | 7.03 ± 0.00 | 6.70 ± 0.01 | 7.25 ± 0.07 | 6.95 ± 0.02 | 6.98 ± 0.01 | 6.98 ± 0.01 |
| ACUF648 | OD | 0.356 ± 0.001 | 0.351 ± 0.005 | 0.352 ± 0.002 | 0.292 ± 0.005 | 0.310 ± 0.001 | 0.303 ± 0.011 | 0.352 ± 0.006 |
| | pH | 7.02 ± 0.01 | 6.98 ± 0.01 | 6.80 ± 0.14 | 7.00 ± 0.01 | 6.98 ± 0.00 | 6.99 ± 0.01 | 7.02 ± 0.01 |
| ACUF731 | OD | 0.321 ± 0.005 | 0.299 ± 0.005 | 0.317 ± 0.020 | 0.364 ± 0.011 | 0.313 ± 0.005 | 0.307 ± 0.006 | 0.356 ± 0.001 |
| | pH | 6.99 ± 0.01 | 7.00 ± 0.00 | 6.95 ± 0.07 | 6.87 ± 0.01 | 6.94 ± 0.01 | 6.95 ± 0.00 | 7.02 ± 0.01 |
| ACUF551 | OD | 0.360 ± 0.006 | 0.328 ± 0.005 | 0.324 ± 0.001 | 0.327 ± 0.005 | 0.313 ± 0.021 | 0.337 ± 0.016 | 0.343 ± 0.006 |
| | pH | 6.97 ± 0.02 | 6.98 ± 0.01 | 6.95 ± 0.07 | 6.89 ± 0.02 | 6.94 ± 0.00 | 6.96 ± 0.01 | 6.99 ± 0.01 |

| Start pH 6.5 | | Time 0 | Week 1 | Week 2 | Week 3 | Week 4 | Week 5 | Week 6 |
|---|---|---|---|---|---|---|---|---|
| IPPAS P507 | OD | 0.388 ± 0.006 | 0.262 ± 0.018 | 0.264 ± 0.052 | 0.252 ± 0.068 | 0.356 ± 0.045 | 0.422 ± 0.119 | 0.668 ± 0.136 |
| | pH | 6.51 ± 0.00 | 6.40 ± 0.06 | 6.33 ± 0.04 | 6.27 ±0.17 | 6.19 ± 0.14 | 5.98 ± 0.21 | 5.68 ± 0.57 |
| ACUF769 | OD | 0.353 ± 0.001 | 0.505 ± 0.094 | 0.681 ± 0.029 | 1.222 ± 0.059 | 2.077 ± 0.111 | 3.930 ± 0.113 | 5.460 ± 0.325 |
| | pH | 6.46 ± 0.01 | 6.05 ± 0.11 | 4.76 ± 0.62 | 3.16 ± 0.11 | 2.80 ± 0.08 | 2.53 ± 0.05 | 2.33 ± 0.03 |
| ACUF722 | OD | 0.365 ± 0.007 | 0.518 ± 0.017 | 0.614 ± 0.023 | 0.827 ± 0.052 | 1.318 ± 0.003 | 2.08 ± 0.035 | 3.005 ± 0.049 |
| | pH | 6.50 ± 0.04 | 6.12 ± 0.04 | 4.26 ± 0.13 | 3.05 ± 0.04 | 2.74 ± 0.02 | 2.47 ± 0.01 | 2.25 ± 0.00 |

| | | | | | | | | |
|---|---|---|---|---|---|---|---|---|
| CloneT18 | OD | 0.372 ± 0.021 | 0.354 ± 0.073 | 0.702 ± 0.006 | 0.748 ± 0.046 | 1.107 ± 0.052 | 2.380 ± 0.042 | 4.160 ± 0.212 |
| | pH | 6.51 ± 0.09 | 6.29 ± 0.00 | 5.33 ± 0.32 | 3.34 ± 0.16 | 2.81 ± 0.08 | 2.55 ± 0.08 | 2.37 ± 0.06 |
| ACUF773 | OD | 0.382 ± 0.001 | 0.735 ± 0.013 | 1.045 ± 0.052 | 1.331 ± 0.064 | 2.042 ± 0.048 | 3.380 ± 0.007 | 5.175 ± 0.120 |
| | pH | 6.55 ± 0.01 | 5.86 ± 0.04 | 3.39 ± 0.45 | 2.90 ± 0.06 | 2.66 ± 0.03 | 2.44 ± 0.02 | 2.17 ± 0.01 |
| ACUF648 | OD | 0.433 ± 0.003 | 0.545 ± 0.011 | 1.342 ± 0.011 | 2.702 ± 0.054 | 4.815 ± 0.092 | 6.745 ± 0.064 | 11.730 ± 0.099 |
| | pH | 6.38 ± 0.01 | 5.67 ± 0.01 | 3.42 ± 0.06 | 2.81 ± 0.30 | 2.77 ± 0.02 | 2.56 ± 0.04 | 2.42 ± 0.01 |
| ACUF731 | OD | 0.324 ± 0.004 | 0.184 ± 0.010 | 0.379 ± 0.001 | 0.877 ± 0.000 | 2.214 ± 0.000 | 4.890 ± 0.099 | 14.863 ± 0.308 |
| | pH | 6.58 ± 0.04 | 6.48 ± 0.03 | 6.14 ± 0.17 | 4.64 ± 0.00 | 3.00 ± 0.01 | 2.62 ± 0.04 | 2.22 ± 0.06 |
| ACUF551 | OD | 0.305 ± 0.028 | 0.721 ± 0.090 | 1.556 ± 0.000 | 2.492 ± 0.000 | 6.910 ± 0.042 | 11.200 ± 0.212 | 17.583 ± 0.513 |
| | pH | 6.48 ± 0.03 | 6.09 ± 0.13 | 3.54 ± 0.02 | 3.00 ± 0.00 | 2.72 ± 0.03 | 2.40 ± 0.05 | 2.16 ± 0.14 |

| **Start pH 6** | | **Time 0** | **Week 1** | **Week 2** | **Week 3** | **Week 4** | **Week 5** | **Week 6** |
|---|---|---|---|---|---|---|---|---|
| IPPAS P507 | OD | 0.349 ± 0.011 | 0.853 ± 0.033 | 1.568 ± 0.017 | 2.644 ± 0.057 | 4.030 ± 0.057 | 5.845 ± 0.106 | 8.670 ± 0.156 |
| | pH | 5.88 ± 0.03 | 3.89 ± 0.29 | 3.08 ± 0.15 | 2.77 ± 0.12 | 2.58 ± 0.11 | 2.43 ± 0.11 | 2.25 ± 0.14 |
| ACUF769 | OD | 0.285 ± 0.013 | 0.587 ± 0.024 | 1.266 ± 0.096 | 2.753 ± 0.117 | 6.021 ± 0.001 | 9.650 ± 0.000 | 14.820 ± 0.000 |
| | pH | 5.83 ± 0.01 | 3.52 ± 0.00 | 2.90 ± 0.02 | 2.58 ± 0.06 | 2.34 ± 0.00 | 2.14 ± 0.02 | 1.84 ± 0.03 |
| ACUF722 | OD | 0.238 ± 0.052 | 0.562 ± 0.011 | 0.856 ± 0.051 | 1.592 ± 0.028 | 2.483 ± 0.152 | 2.818 ± 0.236 | 3.745 ± 0.148 |
| | pH | 5.81 ± 0.01 | 3.76 ± 0.00 | 2.99 ± 0.13 | 2.68 ± 0.14 | 2.47 ± 0.11 | 2.32 ± 0.09 | 2.10 ± 0.14 |
| CloneT18 | OD | 0.271 ± 0.036 | 0.430 ± 0.045 | 0.636 ± 0.006 | 1.524 ± 0.037 | 3.860 ± 0.071 | 6.673 ± 0.046 | 8.196 ± 0.303 |
| | pH | 5.86 ± 0.01 | 4.08 ± 0.30 | 3.18 ± 0.08 | 2.81 ± 0.04 | 2.57 ± 0.02 | 2.34 ± 0.03 | 2.16 ± 0.06 |
| ACUF773 | OD | 0.309 ± 0.010 | 0.752 ± 0.003 | 1.263 ± 0.061 | 2.565 ± 0.147 | 4.890 ± 0.268 | 7.500 ± 0.198 | 8.950 ± 0.028 |
| | pH | 5.79 ± 0.01 | 3.40 ± 0.08 | 2.88 ± 0.06 | 2.65 ± 0.08 | 2.50 ± 0.06 | 2.35 ± 0.08 | 2.33 ± 0.00 |
| ACUF648 | OD | 0.354 ± 0.001 | 0.693 ± 0.063 | 1.769 ± 0.078 | 3.208 ± 0.060 | 4.980 ± 0.014 | 8.140 ± 0.095 | 14.240 ± 0.198 |
| | pH | 5.65 ± 0.25 | 3.87 ± 0.44 | 3.07 ± 0.09 | 2.84 ± 0.05 | 2.62 ± 0.05 | 2.46 ± 0.04 | 2.30 ± 0.01 |

| | | | | | | | | |
|---|---|---|---|---|---|---|---|---|
| ACUF731 | OD | 0.322 ± 0.023 | 0.729 ± 0.001 | 1.423 ± 0.086 | 1.947 ± 0.030 | 5.830 ± 0.121 | 9.520 ± 0.409 | 12.310 ± 0.592 |
| | pH | 6.17 ± 0.71 | 3.61 ± 0.08 | 3.20 ± 0.07 | 2.88 ± 0.07 | 2.67 ± 0.08 | 2.49 ± 0.11 | 2.31 ± 0.12 |
| ACUF551 | OD | 0.354 ± 0.003 | 0.574 ± 0.013 | 1.556 ± 0.079 | 2.928 ± 0.040 | 4.230 ± 0.106 | 7.510 ± 0.099 | 13.920 ± 0.283 |
| | pH | 5.53 ± 0.25 | 3.61 ± 0.01 | 3.02 ± 0.04 | 2.83 ± 0.03 | 2.60 ± 0.03 | 2.43 ± 0.03 | 2.25 ± 0.04 |
| **Start pH 5** | | **Time 0** | **Week 1** | **Week 2** | **Week 3** | **Week 4** | **Week 5** | **Week 6** |
| IPPAS P507 | OD | 0.367 ± 0.027 | 0.984 ± 0.065 | 2.021 ± 0.021 | 3.580 ± 0.071 | 6.610 ± 0.042 | 16.080 ± 0.057 | 18.140 ± 0.141 |
| | pH | 5.08 ± 0.04 | 3.13 ± 0.19 | 2.80 ± 0.10 | 2.47 ± 0.13 | 2.32 ± 0.16 | 2.08 ± 0.24 | 2.16 ± 0.00 |
| ACUF769 | OD | 0.279 ± 0.030 | 0.401 ± 0.058 | 1.140 ± 0.023 | 1.761 ± 0.055 | 3.705 ± 0.049 | 6.880 ± 0.127 | 11.040 ± 0.170 |
| | pH | 5.03 ± 0.05 | 3.41 ± 0.01 | 3.04 ± 0.06 | 2.77 ± 0.01 | 2.58 ± 0.01 | 2.42 ± 0.01 | 2.14 ± 0.04 |
| ACUF722 | OD | 0.252 ± 0.028 | 0.610 ± 0.008 | 1.191 ± 0.016 | 1.880 ± 0.064 | 2.705 ± 0.035 | 2.602 ± 0.059 | 4.925 ± 0.134 |
| | pH | 4.88 ± 0.08 | 3.28 ± 0.04 | 2.93 ± 0.02 | 2.54 ± 0.04 | 2.43 ± 0.07 | 2.26 ± 0.03 | 2.16 ± 0.09 |
| CloneT18 | OD | 0.366 ± 0.003 | 0.593 ± 0.008 | 1.201 ± 0.078 | 1.751 ± 0.064 | 3.360 ± 0.113 | 6.520 ± 0.014 | 10.965 ± 0.021 |
| | pH | 4.83 ± 0.004 | 3.71 ± 0.07 | 3.19 ± 0.13 | 2.80 ± 0.11 | 2.56 ± 0.07 | 2.32 ± 0.00 | 2.00 ± 0.01 |
| ACUF773 | OD | 0.274 ± 0.010 | 0.860 ± 0.074 | 1.589 ± 0.038 | 3.123 ± 0.011 | 5.980 ± 0.141 | 8.030 ± 0.071 | 13.930 ± 0.042 |
| | pH | 4.70 ± 0.02 | 3.12 ± 0.03 | 2.81 ± 0.04 | 2.53 ± 0.01 | 2.46 ± 0.03 | 2.24 ± 0.05 | 2.14 ± 0.03 |
| ACUF648 | OD | 0.391 ± 0.004 | 1.163 ± 0.033 | 1.902 ± 0.040 | 3.260 ± 0.092 | 5.535 ± 0.021 | 13.760 ± 0.028 | 12.350 ± 0.071 |
| | pH | 5.03 ± 0.18 | 3.27 ± 0.01 | 2.89 ± 0.08 | 2.75 ± 0.06 | 2.56 ± 0.05 | 2.44 ± 0.05 | 2.28 ± 0.07 |
| ACUF731 | OD | 0.321 ± 0.019 | 0.796 ± 0.038 | 1.443 ± 0.010 | 2.718 ± 0.053 | 4.740 ± 0.042 | 7.425 ± 0.049 | 10.523 ± 0.081 |
| | pH | 4.84 ± 0.23 | 3.44 ± 0.11 | 3.16 ± 0.06 | 2.86 ± 0.07 | 2.69 ± 0.07 | 2.55 ± 0.09 | 2.22 ± 0.02 |
| ACUF551 | OD | 0.340 ± 0.001 | 0.810 ± 0.076 | 1.950 ± 0.034 | 2.422 ± 0.031 | 6.010 ± 0.099 | 7.925 ± 0.078 | 14.450 ± 0.212 |
| | pH | 5.12 ± 0.25 | 3.35 ± 0.05 | 2.94 ± 0.04 | 2.81 ± 0.08 | 2.55 ± 0.08 | 2.40 ± 0.10 | 2.27 ± 0.12 |
| **Start pH 1.5** | | **Time 0** | **Week 1** | **Week 2** | **Week 3** | **Week 4** | **Week 5** | **Week 6** |
| IPPAS P507 | OD | 0.361 ± 0.004 | 1.002 ± 0.068 | 2.102 ± 0.048 | 3.655 ± 0.085 | 6.925 ± 0.092 | 11.450 ± 0.156 | 19.670 ± 0.268 |
| | pH | 1.29 ± 0.00 | 1.12 ± 0.01 | 1.24 ± 0.03 | 1.57 ± 0.00 | 1.11 ± 0.05 | 1.06 ± 0.07 | 1.13 ± 0.10 |

| | | | | | | | | |
|---|---|---|---|---|---|---|---|---|
| ACUF769 | OD | 0.396 ± 0.006 | 0.808 ± 0.003 | 1.768 ± 0.130 | 3.278 ± 0.011 | 5.750 ± 0.028 | 7.835 ± 0.134 | 12.620 ± 0.113 |
| | pH | 1.42 ± 0.00 | 1.23 ± 0.01 | 1.44 ± 0.01 | 1.72 ± 0.01 | 1.26 ± 0.02 | 1.22 ± 0.01 | 1.33 ± 0.04 |
| ACUF722 | OD | 0.383 ± 0.008 | 0.842 ± 0.045 | 1.910 ± 0.088 | 3.475 ± 0.114 | 6.000 ± 0.085 | 8.503 ± 0.000 | 16.240 ± 0.000 |
| | pH | 1.30 ± 0.00 | 1.13 ± 0.01 | 1.29 ± 0.00 | 1.76 ± 0.04 | 1.12 ± 0.00 | 1.13 ± 0.02 | 1.15 ± 0.01 |
| CloneT18 | OD | 0.405 ± 0.025 | 0.629 ± 0.007 | 1.394 ± 0.059 | 2.234 ± 0.054 | 4.470 ± 0.057 | 7.110 ± 0.085 | 14.010 ± 0.127 |
| | pH | 1.30 ± 0.02 | 1.10 ± 0.00 | 1.34 ± 0.04 | 1.82 ± 0.02 | 1.14 ± 0.01 | 1.12 ± 0.01 | 1.24 ± 0.01 |
| ACUF773 | OD | 0.382 ± 0.009 | 0.667 ± 0.001 | 1.574 ± 0.054 | 2.873 ± 0.060 | 5.120 ± 0.071 | 6.990 ± 0.141 | 13.070 ± 0.127 |
| | pH | 1.33 ± 0.04 | 1.12 ± 0.04 | 1.36 ± 0.03 | 1.80 ± 0.11 | 1.17 ± 0.01 | 1.21 ± 0.02 | 1.21 ± 0.01 |
| ACUF648 | OD | 0.404 ± 0.008 | 1.263 ± 0.055 | 2.504 ± 0.034 | 4.218 ± 0.145 | 7.800 ± 0.099 | 13.540 ± 0.113 | 24.010 ± 0.014 |
| | pH | 1.49 ± 0.01 | 1.38 ± 0.03 | 1.54 ± 0.06 | 1.91 ± 0.02 | 1.30 ± 0.03 | 1.26 ± 0.01 | 1.18 ± 0.05 |
| ACUF731 | OD | 0.394 ± 0.011 | 1.101 ± 0.075 | 2.130 ± 0.031 | 3.500 ± 0.134 | 7.07 ± 0.014 | 9.585 ± 0.233 | 19.060 ± 0.057 |
| | pH | 1.33 ± 0.01 | 1.18 ± 0.04 | 1.35 ± 0.01 | 1.83 ± 0.11 | 1.19 ± 0.01 | 1.14 ± 0.09 | 1.15 ± 0.016 |
| ACUF551 | OD | 0.401 ± 0.016 | 0.835 ± 0.038 | 1.918 ± 0.025 | 4.015 ± 0.035 | 6.305 ± 0.092 | 9.890 ± 0.071 | 15.860 ± 0.170 |
| | pH | 1.46 ± 0.01 | 1.49 ± 0.02 | 1.37 ± 0.01 | 1.38 ± 0.01 | 1.47 ± 0.01 | 1.29 ± 0.01 | 1.20 ± 0.04 |
