# Supplementary figures and images for: Prevalent pH Controls the Capacity of Galdieria maxima to Use Ammonia and Nitrate as a Nitrogen Source
Source: Plants (Basel). 2020 Feb 11;9(2):232. doi: 10.3390/plants9020232 (PMC7076501; doi:10.3390/plants9020232)

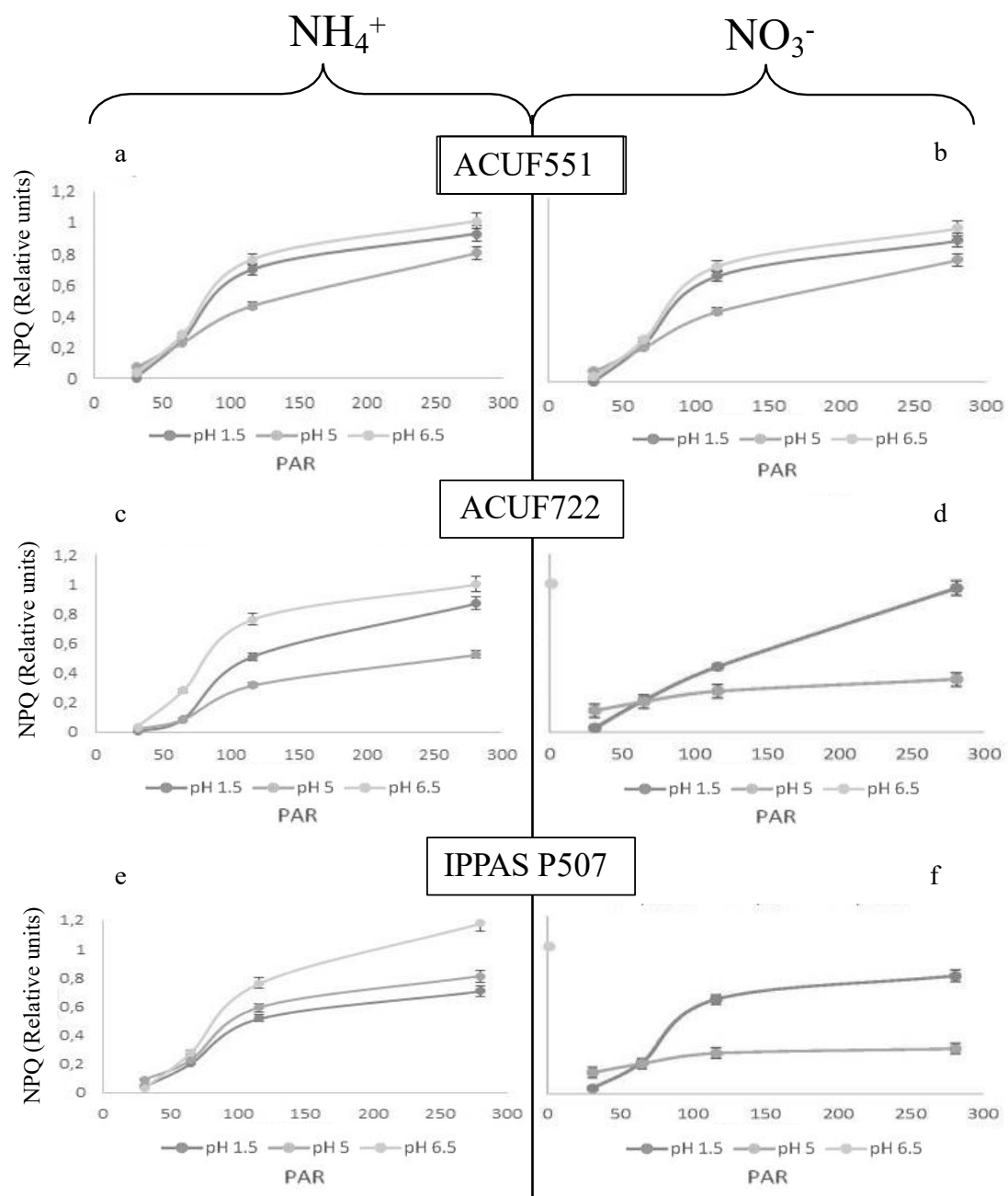

Supplement: Supplementary file 1 [file plants-09-00232-s001.zip › Supplementary materials/FigS4.pdf]

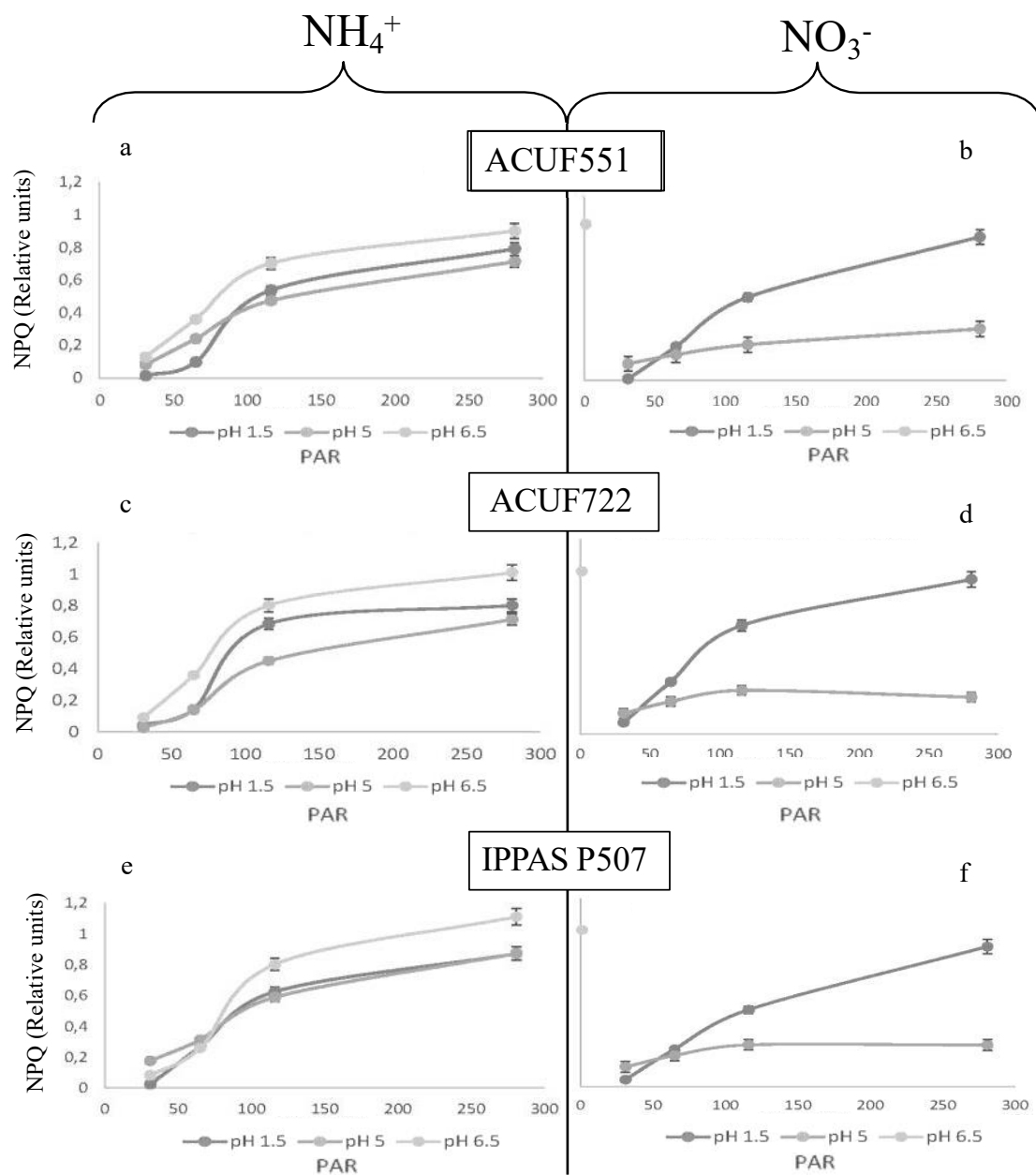

Supplement: Supplementary file 1 [file plants-09-00232-s001.zip › Supplementary materials/FigS5.pdf]

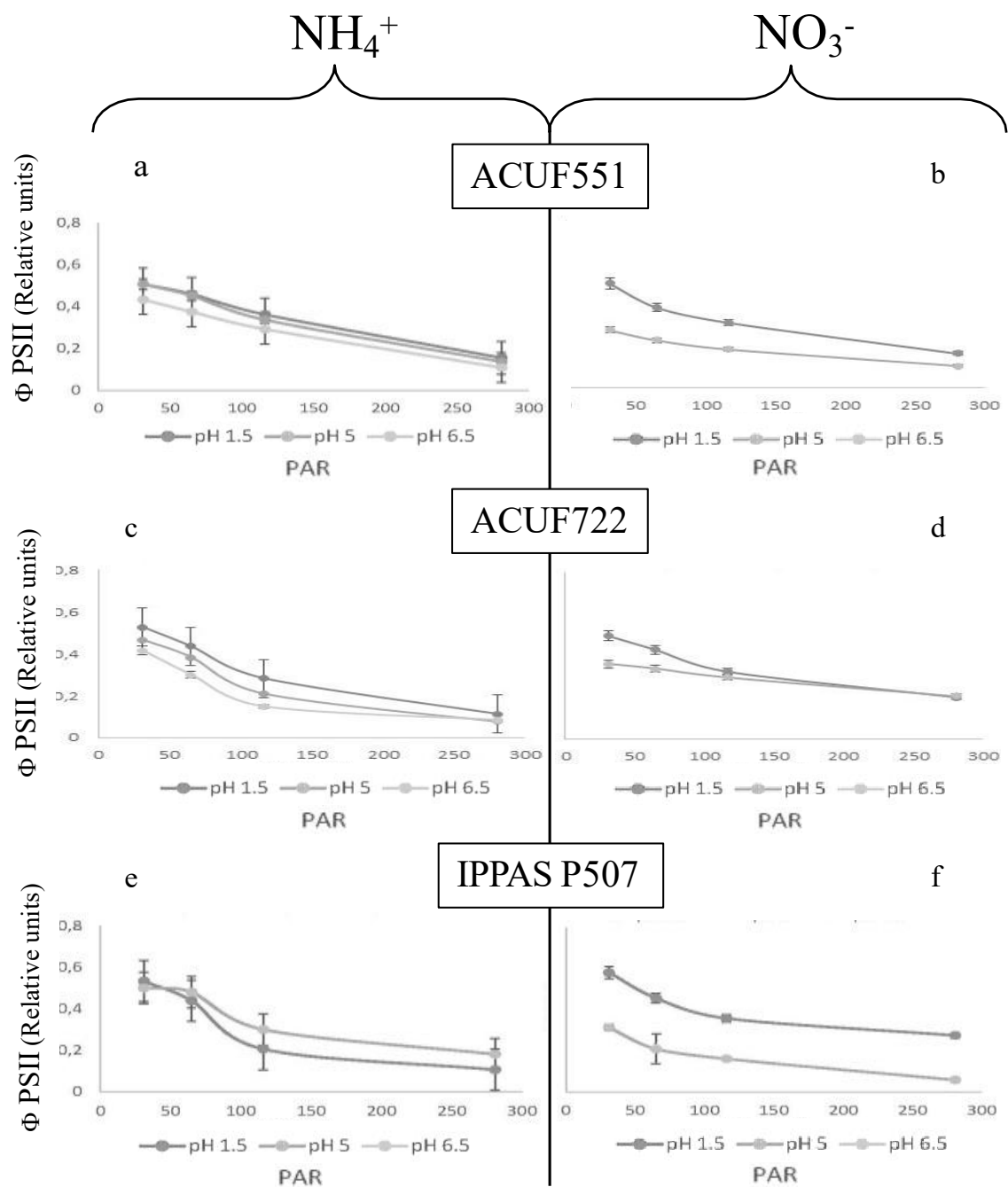

Supplement: Supplementary file 1 [file plants-09-00232-s001.zip › Supplementary materials/FigS7.pdf]

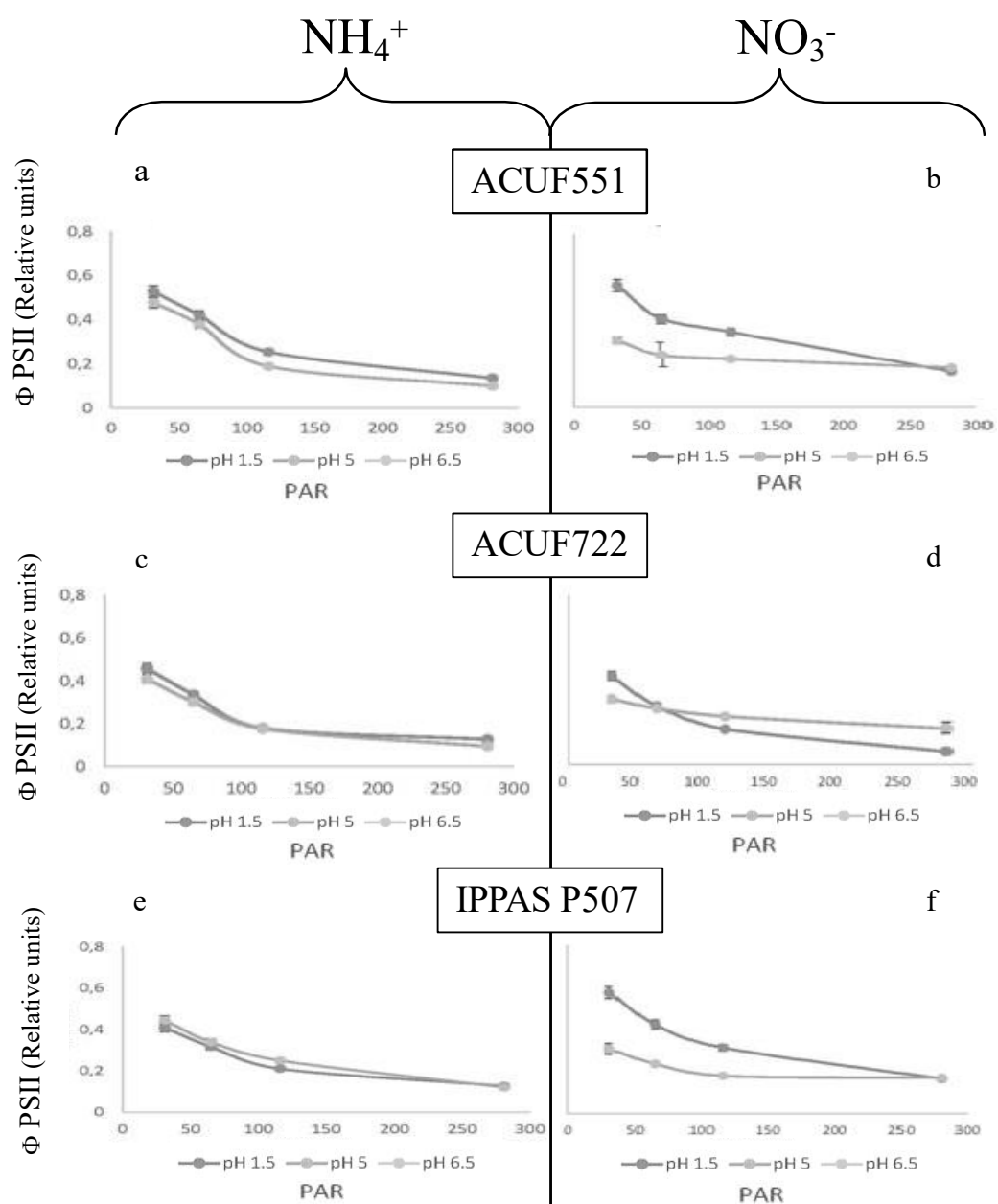

Supplement: Supplementary file 1 [file plants-09-00232-s001.zip › Supplementary materials/FigS6.pdf]

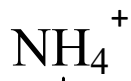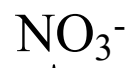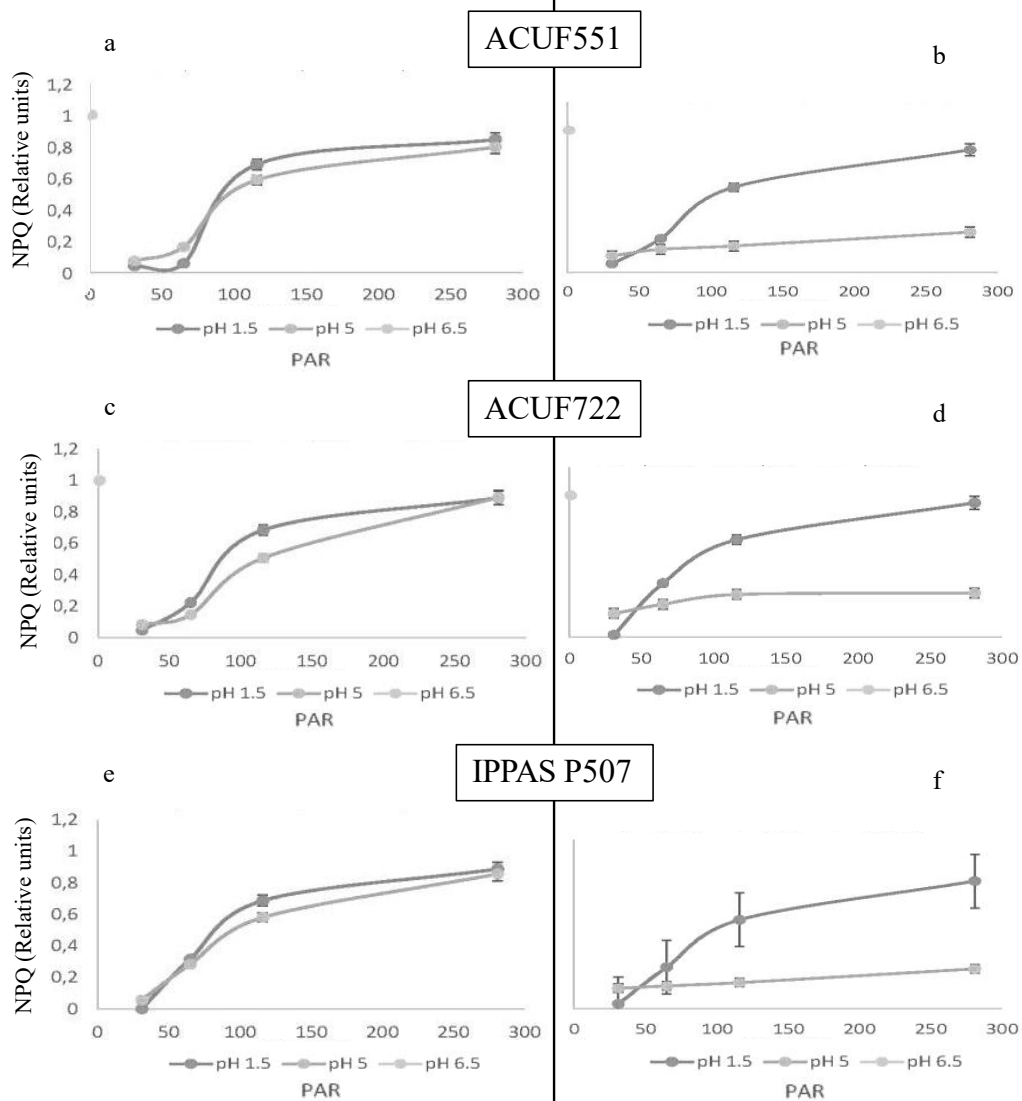

Supplement: Supplementary file 1 [file plants-09-00232-s001.zip › Supplementary materials/FigS2.pdf]

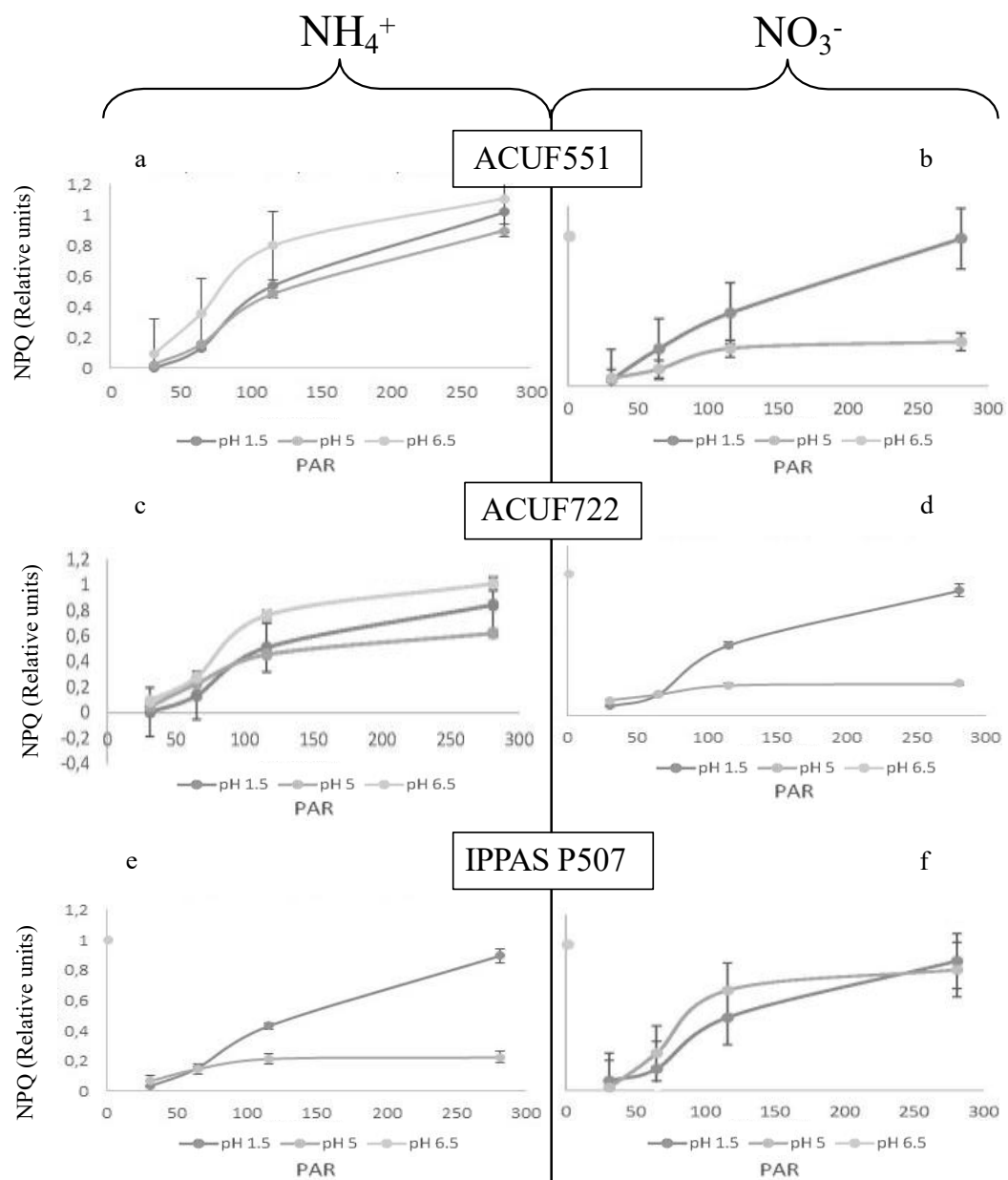

Supplement: Supplementary file 1 [file plants-09-00232-s001.zip › Supplementary materials/FigS3.pdf]

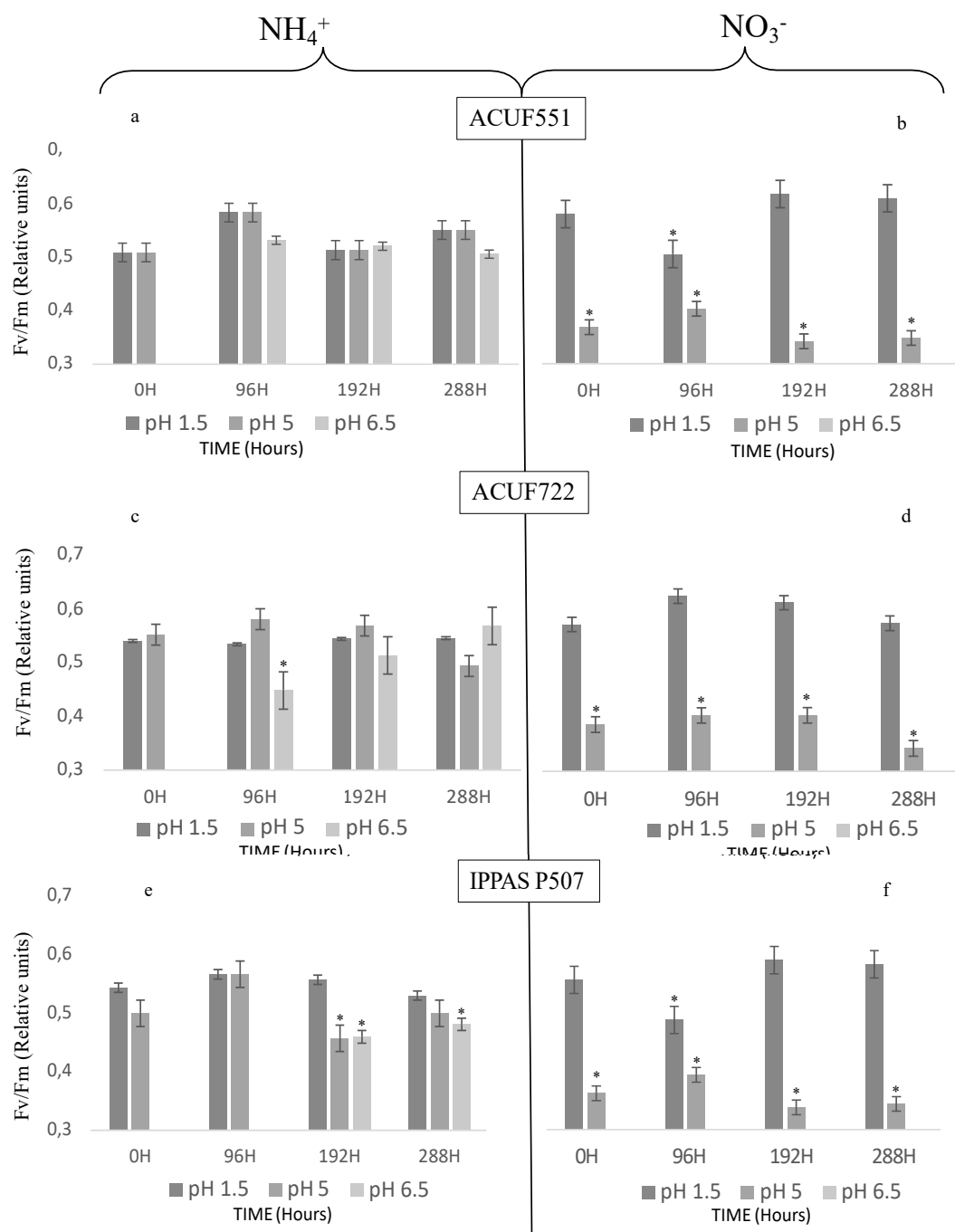

Supplement: Supplementary file 1 [file plants-09-00232-s001.zip › Supplementary materials/FigS1.pdf]

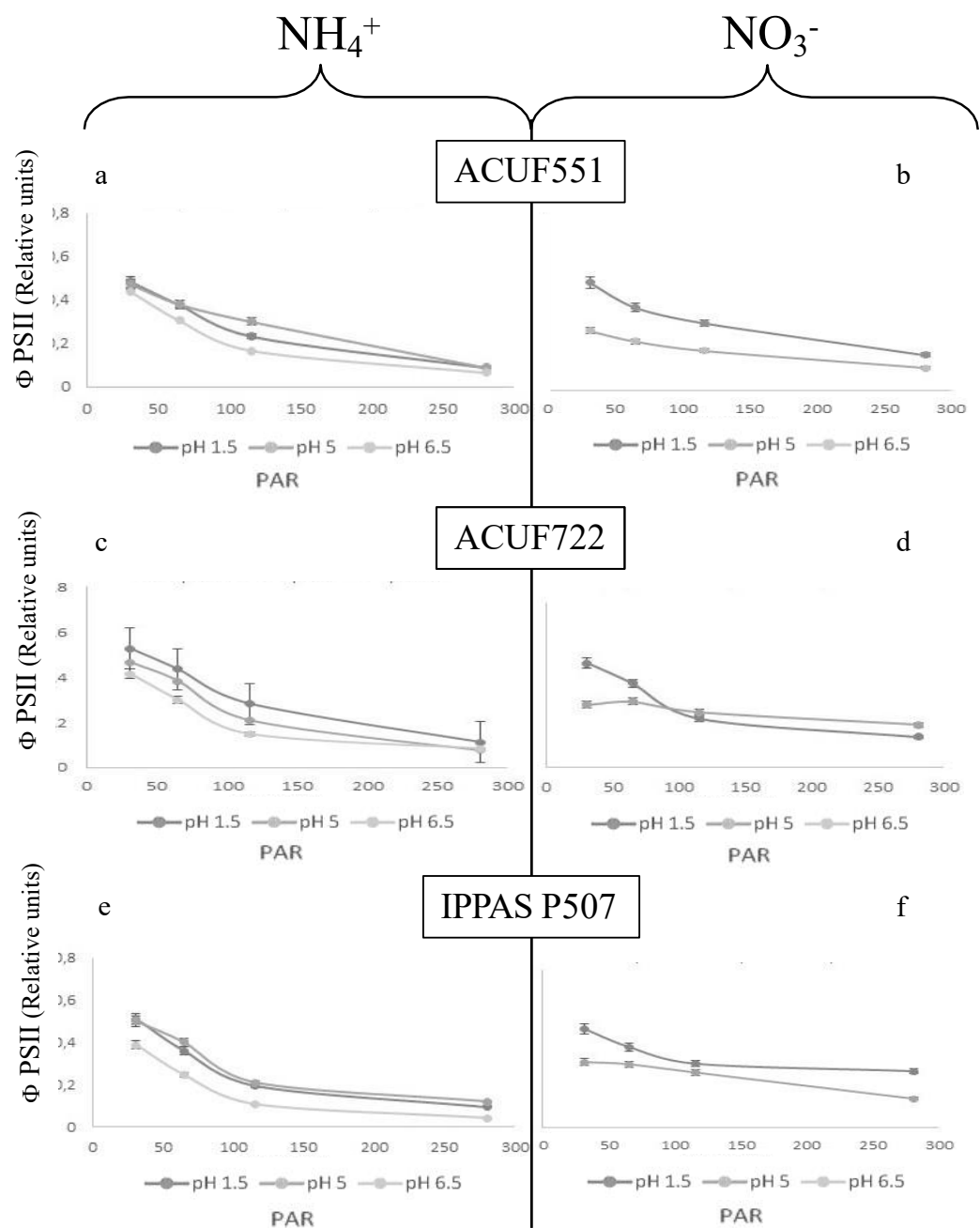

Supplement: Supplementary file 1 [file plants-09-00232-s001.zip › Supplementary materials/FigS8.pdf]

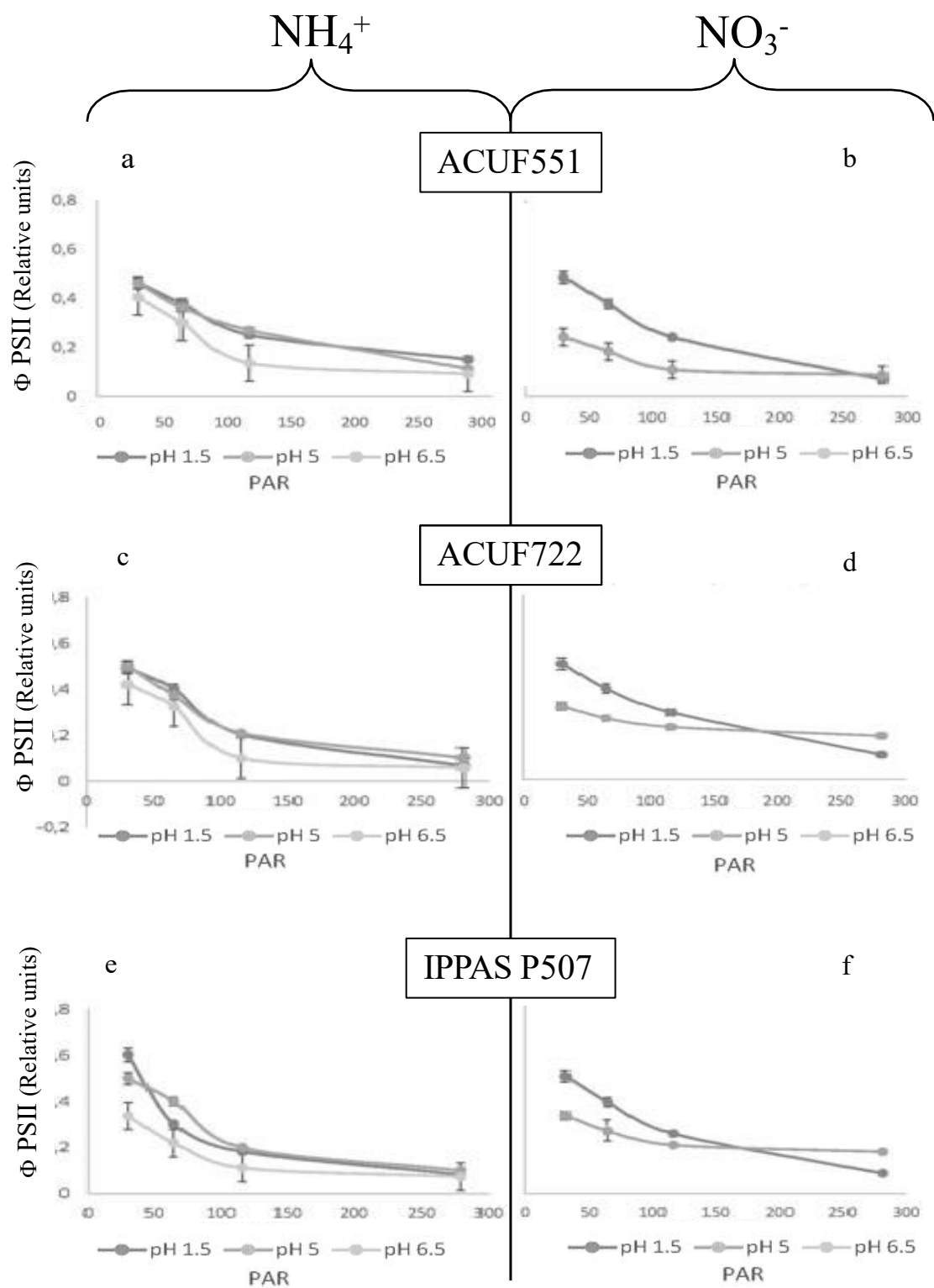

Supplement: Supplementary file 1 [file plants-09-00232-s001.zip › Supplementary materials/FigS9.pdf]
